# AINTEGUMENTA and AINTEGUMENTA-LIKE6 directly regulate floral homeotic, growth, and vascular development genes in young Arabidopsis flowers

**DOI:** 10.1093/jxb/erab223

**Published:** 2021-05-20

**Authors:** Beth A Krizek, Alexis T Bantle, Jorman M Heflin, Han Han, Nowlan H Freese, Ann E Loraine

**Affiliations:** 1 Department of Biological Sciences, University of South Carolina, Columbia, SC, USA; 2 Department of Bioinformatics and Genomics, University of North Carolina at Charlotte, Charlotte, NC, USA; 3 University College Dublin, Ireland

**Keywords:** AINTEGUMENTA (ANT), AINTEGUMENTA-LIKE6 (AIL6), *Arabidopsis thaliana*, ChIP-Seq, floral homeotic genes, floral organ identity, flower development, organ growth, organ size, vascular development

## Abstract

Arabidopsis flower primordia give rise to organ primordia in stereotypical positions within four concentric whorls. Floral organ primordia in each whorl undergo distinct developmental programs to become one of four organ types (sepals, petals, stamens, and carpels). The Arabidopsis transcription factors AINTEGUMENTA (ANT) and AINTEGUMENTA-LIKE6 (AIL6) are required for correct positioning of floral organ initiation, contribute to the specification of floral organ identity, and regulate the growth and morphogenesis of developing floral organs. To gain insight into the molecular mechanisms by which ANT and AIL6 contribute to floral organogenesis, we identified the genome-wide binding sites of both ANT and AIL6 in stage 3 flower primordia, the developmental stage at which sepal primordia become visible and class B and C floral homeotic genes are first expressed. AIL6 binds to a subset of ANT sites, suggesting that AIL6 regulates some but not all of the same target genes as ANT. ANT- and AIL6-binding sites are associated with genes involved in many biological processes related to meristem and flower organ development. Comparison of genes associated with both ANT and AIL6 ChIP-Seq peaks and those differentially expressed after perturbation of ANT and/or AIL6 activity identified likely direct targets of ANT and AIL6 regulation. These include class B and C floral homeotic genes, growth regulatory genes, and genes involved in vascular development.

## Teaser

Arabidopsis ANT and AIL6 directly regulate genes involved in different aspects of early flower development including genes that specify floral organ identity and those that regulate growth and vascular development.

## Introduction

Flowers have long fascinated humans for both their beauty and their morphological diversity. In addition, flowers are of practical importance since they contribute to human nutrition in the form of fruits, seeds, and grains. In *Arabidopsis thaliana*, flowers arise iteratively from the periphery of the inflorescence meristem, a dome-shaped structure at the apex of the plant. Auxin accumulation within these cells activates MONOPTEROS/AUXIN RESPONSE FACTOR5 (MP/ARF5), a transcription factor that up-regulates expression of the floral meristem identity gene *LEAFY* (*LFY*) as well as two *AINTEGUMENTA-LIKE/PLETHORA* (*AIL/PLT*) genes, *AINTEGUMENTA* (*ANT*) and *AIL6/PLT3*, to promote flower primordia identity and outgrowth (Yamaguchi *et al.*, 2013). ANT and AIL6, two members of the larger APETALA2/ETHYLENE RESPONSE FACTOR (AP2/ERF) transcription factor family, and LFY, a plant-specific transcription factor, act early to establish flower primordia and later to promote their continued development with the specification and elaboration of different floral organ types (Weigel *et al.*, 1992; Weigel and Meyerowitz, 1993; Elliott *et al.*, 1996; Klucher *et al.*, 1996; Krizek, 2009; Yamaguchi *et al.*, 2016).

Floral meristems give rise to floral organ primordia at precise positions within four concentric whorls. Floral organ primordia adopt one of four fates (sepal, petal, stamen, or carpel) based on their relative position within the developing flower. Positional information within floral primordia is conveyed through distinct combinations of floral organ identity gene activities in each whorl of the flower, as summarized in the ABCE model (reviewed in Wellmer *et al.*, 2014). In the outermost whorl one, the class A [*APETALA1* (*AP1*) and *APETALA2* (*AP2*)] and E [*SEPALLATA1–4* (*SEP1–SEP4*)] genes specify sepal identity. In whorl two, the class A, B [*APETALA3* (*AP3*) and *PISTILLATA* (*PI*)], and E genes specify petal identity. In whorl three, class B, C [*AGAMOUS* (*AG*)], and E genes specify stamen identity, and in whorl four, class C and E genes specify carpel identity. Mutations in class A, B, and C genes result in homeotic changes in floral organ identity in two adjacent whorls of the flower. Thus, these genes are also referred to as floral homeotic genes. All of the class A, B, C, and E genes, except the class A gene *AP2*, encode MADS domain transcription factors (Yanofsky *et al.*, 1990; Jack *et al.*, 1992; Mandel *et al.*, 1992; Goto and Meyerowitz, 1994; Pelaz *et al.*, 2000; Ditta *et al.*, 2004). AP2 is a member of the AP2/ERF transcription factor family which also includes the AIL/PLT proteins ANT and AIL6 (Jofuku *et al.*, 1994).

Both *lfy* single mutants and *ant ail6* double mutants display loss of floral organ identities but do not show homeotic transformations in organ identity as described for mutations in the class A, B, and C genes (Weigel *et al.*, 1992; Krizek, 2009). *lfy* flowers consist primarily of leaf-like and carpel-like organs, and exhibit reduced expression of class B and C genes (Weigel and Meyerowitz, 1993). Broadly expressed LFY acts with region-specific cofactors to directly activate expression of class B and C genes in stage 3 flowers (Lee *et al.*, 1997; Busch *et al.*, 1999; Lenhard *et al.*, 2001; Lohmann *et al.*, 2001; Lamb *et al.*, 2002; Liu *et al.*, 2009). *ant ail6* double mutants produce flowers with sepals, filaments, stamenoid organs, unfused carpel valves, and structures that do not resemble any wild-type floral organs (Krizek, 2009). *ANT* and *AIL6* act redundantly to promote petal, stamen, and carpel identity as these floral organs are present in both *ant* and *ail6* single mutants (Elliott *et al.*, 1996; Klucher *et al.*, 1996; Krizek, 2009). Previous work has shown that expression of the class B genes *AP3* and *PI*, and the class C gene *AG* is reduced in *ant ail6* flowers, but it was not known whether ANT and AIL6 directly activate these genes in stage 3 flowers (Krizek, 2009; Krizek *et al.*, 2016).

In addition to defects in floral organ identity, *ant ail6* flowers make fewer and smaller floral organs that do not arise with regular phyllotaxy (Krizek, 2009). *ANT* plays a more important role than *AIL6* in promoting floral organ growth as *ant* floral organs are reduced in size while *ail6* flowers are normal in appearance (Elliott *et al.*, 1996; Klucher *et al.*, 1996; Krizek, 2009). However, the enhanced growth defects in *ant ail6* double mutant flowers indicate that *AIL6* also contributes to organ growth (Krizek, 2009). Overexpression of either *ANT* or *AIL6* can result in larger flowers, indicating that both genes are sufficient for floral organ growth (Krizek, 1999; Mizukami and Fischer, 2000; Krizek and Eaddy, 2012; Han and Krizek, 2016).

Here we used ChIP in combination with next-generation sequencing (ChIP-Seq) to investigate the molecular means by which ANT and AIL6 regulate early events in flower development. We identified genome-wide binding sites for both ANT and AIL6 in stage 3 flowers, the stage at which sepal primordia are first visible and class B and C gene expression is initiated. Our work reveals that the partial redundancy of *ANT* and *AIL6* results from AIL6 binding a subset of the genomic regions bound by ANT. Both ANT and AIL6 bind genomic loci associated with genes regulating many different developmental processes including radial patterning, polarity specification, boundary formation, floral meristem determinacy, and floral organ morphogenesis. To identify the most likely direct targets of ANT and AIL6 regulation, we compared our ChIP-Seq data with genes previously identified as differentially expressed in either *ant ail6* double mutant inflorescences compared with the wild type or those differentially expressed after induction of ANT activity in steroid-treated *35S:ANT-GR* inflorescences (Krizek *et al.*, 2016, 2020). Furthermore, we investigated how the expression of some of these candidate direct target genes responds to inducible down-regulation of *AIL6* in the *ant* mutant background or inducible down-regulation of *ANT* alone. Our data support a direct role for ANT and AIL6 in activating the floral homeotic genes *AP3* and *AG* in stage 3 flowers, in regulating the expression of four growth regulatory genes: *BIG BROTHER* (*BB*), *ROTUNDIFOLIA 3* (*ROT3*), *ANGUSTIFOLIA3/GRF-INTERACTING FACTOR 1* (*AN3/GIF1*), and *XYLOGLUCAN ENDOTRANSGLUCOSYLASE/HYDROLASE9* (*XTH9*), and in regulating genes associated with vascular development.

## Materials and methods

### Plant materials and growth conditions

*ANT:ANT-VENUS ant-4 AP1:AP1-GR ap1 cal* plants and *AIL6:AIL6-VENUS* plants were described previously (Han and Krizek, 2016; Krizek *et al.*, 2020). *AIL6:AIL6-VENUS* plants were crossed to *AP1:AP1-GR ap1cal* plants. *AP1:AP1-GR ap1 cal*, *ANT:ANT-VENUS ant-4 AP1:AP1-GR ap1 cal*, and *AIL6:AIL6-VENUS AP1:AP1-GR ap1 cal* inflorescences were grown on a soil mixture of Fafard 4P:perlite:vermiculite (8:1:1) in 24 h days at a light intensity of ~160 μmol m^–2^ s^–1^ at 20 °C. An artificial miRNA (amiRNA) that targets AIL6 was designed using http://wmd3.weigelworld.org/cgi-bin/webapp.cgi. A gene fragment containing this AIL6-amiR within the miR319a precursor was synthesized by IDT and cloned into the *Eco*RI/*Bam*HI sites of BJ36_AlcA to create AlcA:AIL6-amiR/BJ36. AlcA:AIL6-amiR was subcloned into the *Not*I site of pMLBart_AlcR to create 35S:ALCR/AlcA:AIL6-amiR/pMLBart. 35S:ALCR/AlcA:AIL6-amiR/pMLBart was transformed into *Agrobacterium* strain ASE, which was then used to transform *ant-4/+* plants. *35S:ALCR/AlcA:AIL6-amiR* transgenic plants were selected for Basta resistance. The first 736 nucleotides of the *ANT* coding region were cloned in the sense and antisense directions in pHannibal using ANTIR-5 and ANTIR-6 (Supplementary Table S1). The ANT-IR fragment was digested from pHannibal with *Bam*HI and cloned into BJ36_AlcA to create AlcA:ANT-IR. AlcA:ANT-IR was subcloned into the *Not*I site of pMLBart_AlcR to create 35S:ALCR/AlcA:ANT-IR/pMLBart. 35S:ALCR/AlcA:ANT-IR/pMLBart was transformed into *Agrobacterium* strain ASE, which was then used to transform L*er* plants. *35S:ALCR/AlcA:ANT-IR* transgenic plants were selected for Basta resistance. *35S:ALCR/AlcA:AIL6-amiR ant-4* and *35S:ALCR/AlcA:ANT-IR* plants were grown on a soil mixture of Fafard 4P:perlite:vermiculite (8:1:1) in 16 h days at a light intensity of ~160 μ mol m^–2^ s^–1^ at 22 °C .

### ChIP-Seq and ChIP-qPCR

Plants for ChIP-Seq and ChIP-qPCR were treated by pipetting a dex (10 μ M dexamethasone+0.015% Silwet) solution onto the inflorescences. ChIP was performed as described previously except that inflorescences were collected 2 d after dex treatment when the tissue consists of stage 3 flowers (Krizek *et al.*, 2020). Primers for ChIP-qPCR are listed in Supplementary Table S1. Fold enrichment was determined relative to a negative control, the transposon *TA3*. Sequencing libraries were prepared from two biological replicates of input and ChIP DNA for *AP1:AP1-GR ap1 cal*, *ANT:ANT-VENUS ant-4 AP1:AP1-GR ap1 cal*, and *AIL6:AIL6-VENUS AP1:AP1-GR ap1 cal* as described previously (Krizek *et al.*, 2020). Sequencing was performed on an Illumina HiSeq 2500 producing 150 base paired-end reads. Sequence reads were aligned to the reference *A. thaliana* genome (version TAIR9, released June 2009) using bowtie2. Examination by eye of the coverage graphs for each chromosome revealed high reproducibility between the two ChIP-Seq replicates. In addition, the input samples closely resembled the control untagged *AP1:AP1-GR ap1 cal* samples. ANT and AIL6 binding peaks were identified using a visual analytics approach within the Integrated Genome Brower (IGB) (Freese *et al.*, 2016). Specifically, coverage graphs were generated for the combined data from the two replicates and normalized. A difference coverage graph was generated by subtracting coverage graphs of the untagged sample (*AP1:AP1-GR ap1 cal*) from the coverage graphs for the tagged samples (*ANT:ANT-VENUS ant-4 AP1:AP1-GR ap1 cal* and *AIL6:AIL6-VENUS AP1:AP1-GR ap1 cal*). Peaks were defined using the thresholding feature. A thresholding value of 2.5 identified 595 peaks for AIL6 and 2631 peaks for ANT in stage 3 flowers. For each identified peak, ChIPpeakAnno was used to identify the gene with the closest transcription start site (TSS) (Zhu *et al.*, 2010). In some cases, a peak located within a gene is associated with two genes if the TSS of an adjacent gene is closer than the TSS of the gene overlapping the peak. Gene Ontology (GO) analyses were performed with AmiGO 2 (http://amigo.geneontology.org/amigo). Enriched GO terms were identified using a Fisher’s exact test of contingency tables followed by Bonferroni correction for multiple hypothesis testing. BEDtools intersect was used to identify overlapping ANT and AIL6 peaks in stage 3 flowers. *De novo* motif discovery was performed with MEME-ChIP (Machanick and Bailey, 2011) searching for five MEME motifs using the Arabidopsis DAP motifs (O’Malley *et al.*, 2016). FIMO was used to locate individual occurrences of MEME-identified motifs within common ANT- and AIL6-binding sites (Grant *et al.*, 2011). The relative position of identified motifs was calculated using the Distance program. Source codes for bioinformatic analyses including the Distance program are available in the project ‘git’ repository (https://bitbucket.org/krizeklab/antail6stage3chipseq/). Venn diagrams were created using Venn Diagram Plotter (https://omics.pnl.gov) created by the Pacific Northwest National Lab.

### RT-qPCR

*35S:AlcR/AlcA:AIL6-amiR ant-4* and *35S:ALCR/AlcA:ANT-IR* plants were mock treated or treated with ethanol vapor by placing 2 ml of water or 2 ml of 100% ethanol in 2 ml centrifuge tubes in half of the pots in a tray. The tray was covered with a plastic dome. Inflorescences were collected at the end of an 8 h (*35S:AlcR/AlcA:AIL6-amiR ant-4*) or 24 h (*35S:ALCR/AlcA:ANT-IR*) treatment. RNA was isolated using an RNeasy Plant Mini Kit (Qiagen) or TRIzol (Life Technologies). Samples isolated with TRIzol were further purified on an RNeasy column (Qiagen) and treated with DNase while on the column. First-strand cDNA synthesis was performed using Quanta qScript cDNA SuperMix (Quanta BioSciences) following the manufacturer’s instructions. Quantitative PCR (qPCR) was performed on a BioRad CFX96 or CFX Connect real-time PCR system using PerfeCTa SYBR Green FastMix for iQ (Quanta BioSciences) and primers listed in Supplementary Table S1. Data analyses were carried out as described previously (Krizek and Eaddy, 2012). At least two biological replicates were analyzed for each experiment.

### Gel mobility shift assays

The gel mobility shift assays were performed as described previously (Nole-Wilson and Krizek, 2000).

### Yeast strain and β-galactosidase assays

A reporter plasmid was made by cloning 76 bp of *AG* intron sequence upstream of the *lacZ* gene in the vector pLacZi (Clontech). The yeast reporter strain was made by integration of the linearized *AG* intron reporter plasmid into the yeast strain YM4271. This new reporter strain was transformed with the previously described ANT/pGAD424 lacking the GAL4 activation domain (Krizek, 2003). Transformants were selected on plates containing synthetic medium lacking leucine. β-Galactosidase assays were performed as described previously (Krizek, 2003).

### Petal size measurements

Petal measurements for mock- and ethanol-treated L*er* and *35S:ALCR/AlcA:ANTIR* were performed on 10–20 petals from different flowers at positions 1–10 on an inflorescence. Petal measurements were performed as described previously (Trost *et al.*, 2014). Petals from stage 13 flowers were removed with forceps and placed on Sellotape. The tape was adhered to a piece of black plexiglass and scanned at a resolution of 3600 dpi in 8-bit grayscale. Petal area, length, and width were determined using Image J software.

## Results

### ChIP-Seq identifies many more ANT-binding peaks than AIL6-binding peaks

To begin to understand the overlapping functions of ANT and AIL6 in early stages of flower development, we performed ChIP-Seq using previously described ANT–VENUS and AIL6–VENUS fusion lines expressed under their respective endogenous promoters in the *AP1:AP1-GR ap1 cal* synchronized floral induction system (O’Maoiléidigh *et al*., 2015; Han and Krizek, 2016; Krizek *et al.*, 2020). The *AP1:AP1-GR ap1 cal* system allows for the collection of inflorescences composed of flowers of a single stage of flower development (O’Maoiléidigh *et al*., 2015). Inflorescences from *AP1:AP1-GR ap1 cal* (no tag), *ANT:ANT-VENUS ant AP1:AP1-GR ap1 cal* (ANT–VENUS), and *AIL6:AIL6-VENUS AP1:AP1-GR ap1 cal* (AIL6–VENUS) plants were collected 2 d after dex treatment when the inflorescences are composed of stage 3 flowers. Peaks were identified using a visual analytic method in IGB (Freese *et al.*, 2016) in which signal from the no tag sample was subtracted from the tagged sample and signals above a threshold value were identified. This method revealed 2631 peaks for ANT–VENUS and 595 peaks for AIL6–VENUS. Visual inspection of the data reveals many overlapping ANT and AIL6 peaks. However, there are many more ANT peaks than AIL6 peaks, and ANT peaks are higher in signal than AIL6 peaks. This suggests that ANT regulates more genes than AIL6 and that there is generally lower occupancy of genomic regions by AIL6 as compared with ANT in the collected tissue. This is consistent with the more important role of ANT as compared with AIL6 in floral organ development (Krizek, 2009). Loss of *ANT* function results in smaller floral organs and slight reductions in floral organ number, while loss of *AIL6* function has no phenotypic consequence (Elliott *et al.*, 1996; Klucher *et al.*, 1996; Krizek, 2009). The lower signal of the majority of AIL6-binding peaks as compared with corresponding ANT peaks may be a consequence of lower levels of AIL6 protein as compared with ANT in developing flowers or from AIL6 having lower affinity for these genomic regions as compared with ANT. In addition, competition between the endogenous AIL6 protein and the transgenic AIL6–VENUS protein may have lowered the ChIP-Seq signal in this experiment compared with that for ANT–VENUS. For the ANT–VENUS line, we used an *ant* mutant background in which there is probably no endogenous ANT protein produced.

**Fig. 1.**
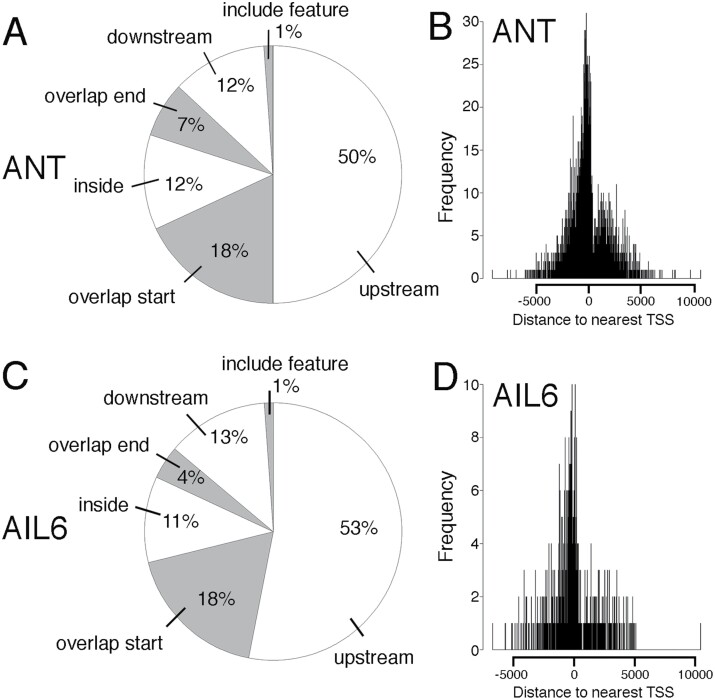
Position of ANT and AIL6 ChIP-Seq peaks relative to the closest gene. (A) Pie chart showing the position of ANT ChIP-Seq binding peaks relative to the closest gene. Approximately half of the peaks are upstream of the closest gene (50%). The remaining peaks either overlap the start of the gene (18.0%), are within the gene (12%), overlap the end of the gene (7%), are downstream of the gene (12%), or overlap the entire gene (1%). (B) Position of ANT-binding peaks relative to the transcriptional start site (TSS) of the closest gene. (C) Pie chart showing the position of AIL6 ChIP-Seq binding peaks relative to the closest gene. Approximately half of the peaks are upstream of the closest gene (53%). The remaining peaks either overlap the start of the gene (18.0%), are within the gene (11%), overlap the end of the gene (4%), are downstream of the gene (13%), or overlap the entire gene (1%). (D) Position of AIL6-binding peaks relative to the TSS of the closest gene.

We used ChIPpeakAnno to identify genes associated with ANT- and AIL6-binding peaks (Zhu *et al.*, 2010). ANT peaks were associated with 2318 unique genes while AIL6 peaks were associated with 592 unique genes (Supplementary Dataset S1). ANT and AIL6 peaks showed similar distributions relative to the positions of genes. For ANT, half of the peaks (50%) are present upstream of the gene, with the remaining peaks either overlapping the start of transcription (18%), within the gene (12%), overlapping the end of transcription (7%), downstream of the gene (12%), or encompassing the gene (1%) (Fig. 1A). For AIL6, over half of the peaks (53%) are present upstream of the gene with the remaining peaks either overlapping the start of transcription (18%), within the gene (11%), overlapping the end of transcription (4%), downstream of the gene (13%), or encompassing the gene (1%) (Fig. 1C). For both ANT and AIL6, the majority of peaks are located within 2.5 kb of the TSS (Fig. 1B, D). The average distance upstream from the TSS was 37 bp for ANT and 90 bp for AIL6.

### GO term enrichment analyses link ANT and AIL6 function with meristem and flower development, hormone physiology, and transcriptional regulation

**Fig. 2.**
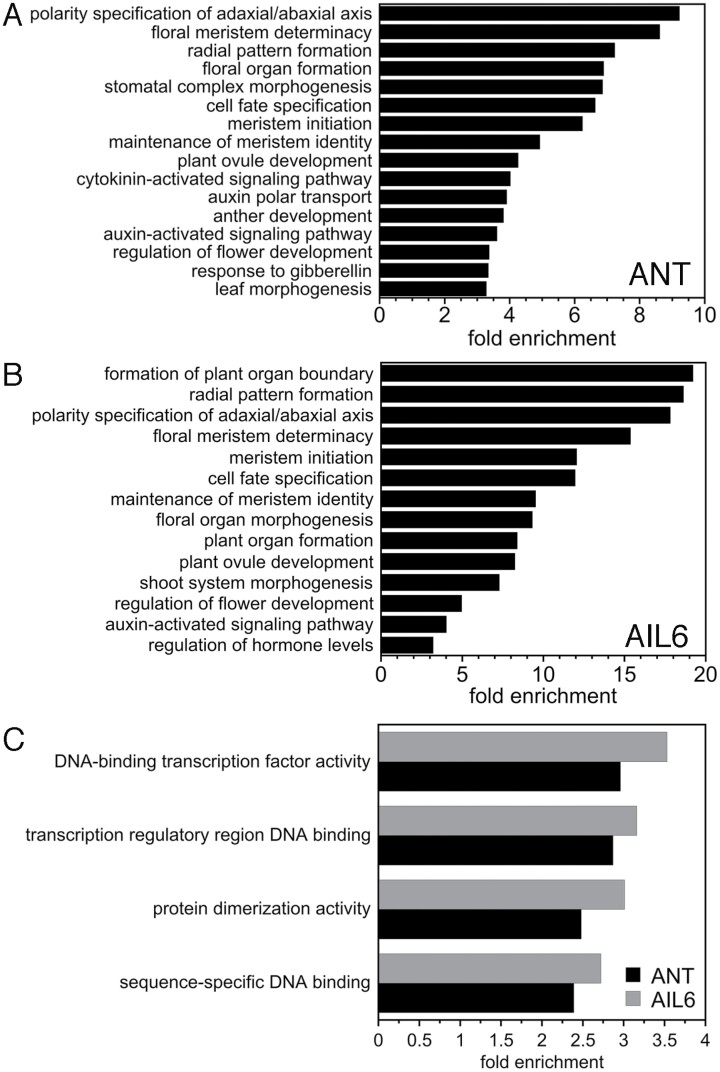
GO enrichment analyses on genes associated with ANT and AIL6 ChIP-Seq binding peaks. (A) Biological process GO terms enriched in genes associated with ANT-binding peaks. (B) Biological process GO terms enriched in genes associated with AIL6-binding peaks. (C) Molecular function GO terms enriched in genes associated with ANT- and AIL6-binding peaks.

GO enrichment analyses identified a number of over-represented terms in the set of genes associated with either ANT or AIL6 peaks (Fig. 2; Supplementary Datasets S2, S3). Over-represented GO biological process terms that were identified for both ANT and AIL6 include many associated with meristem and lateral organ development including: polarity specification of the adaxial/abaxial axis (GO:0009944), floral meristem determinacy (GO:0010582), radial pattern formation (GO:0009956), cell fate specification (GO:0001708), meristem initiation (GO:0010014), maintenance of meristem identity (GO:0010074), plant ovule development (GO:0048481), and regulation of flower development (GO:0009909) (Fig. 2A, B). Other over-represented biological process development-related GO terms for genes associated with ANT-binding peaks include: floral organ formation (GO:0048449), stomatal complex morphogenesis (GO:0010103), anther development (GO:0048653), and leaf morphogenesis (GO:0009965) (Fig. 2A). Additional over-represented GO terms for genes associated with AIL6-binding peaks were: formation of plant organ boundary (GO:0090691), floral organ morphogenesis (GO:0048444), plant organ formation (GO:1905393), and shoot system morphogenesis (GO:0010016) (Fig. 2B).

One over-represented biological process hormone-related GO term associated with both ANT- and AIL6-binding peaks was the auxin-activated signaling pathway (GO:0009734) (Fig. 2A, B). In addition, other over-represented hormone terms for genes associated with ANT peaks include cytokinin-activated signaling pathway (GO:0009736), auxin polar transport (GO:0009926), response to gibberellin (GO:0009739), ethylene-activated signaling pathway (GO:0009873), and response to abscisic acid (GO:0009737) (Fig. 2A, B; Supplementary Dataset S2). These results suggest that both ANT and AIL6 regulate auxin signaling while ANT plays a more general role in mediating multiple hormone signaling pathways and responses.

Several over-represented molecular function GO terms for both ANT and AIL6 are associated with transcriptional regulation. These include DNA-binding transcription factor activity (GO:0003700), transcription regulatory region DNA binding (GO:0044212), protein dimerization activity (GO:0046983), and sequence-specific DNA binding (GO:0043565) (Fig. 2C; Supplementary Datasets S2, S3). ANT and AIL6 bind to numerous genomic regions associated with transcription factors that regulate development.

### AIL6-binding peaks correspond to a subset of ANT peaks

**Fig. 3.**
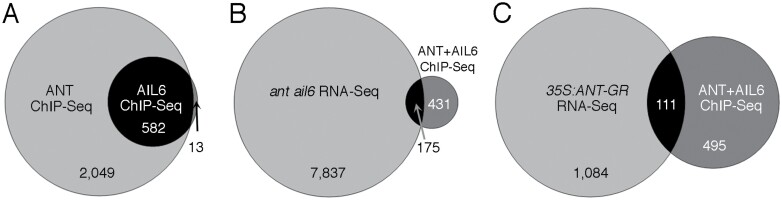
Venn diagrams showing the overlap of ANT and AIL6 ChIP-Seq data and the overlap of genes that are bound by both ANT and AIL6 and differentially expressed in *ant ail6* or *35S:ANT-GR* RNA-Seq experiments. (A) Venn diagram showing the overlap of ANT and AIL6 ChIP-Seq binding peaks. (B) Venn diagram showing the overlap of genes associated with both ANT and AIL6 ChIP-Seq peaks and genes differentially expressed in *ant ail6* compared with L*er* (RNA-Seq). (C) Venn diagram showing the overlap of genes associated with both ANT and AIL6 ChIP-Seq peaks and genes differentially expressed in dex-treated *35S:ANT-GR* compared with mock-treated *35S:ANT-GR* (RNA-Seq).

We determined the degree of overlap between ANT- and AIL6-binding peaks. A total of 582 out of the 595 AIL6 peaks (~98%) overlap at least 50% with an ANT peak (Fig. 3A). For the 13 AIL6 peaks that did not overlap at least 50% with an ANT peak, this was because there was no nearby ANT peak (three), the AIL6 peak was wider with a summit shifted compared with an ANT peak in the same region (three), or there was an overlapping ANT peak but its height was below the 2.5 threshold value used for peak identification (seven) (Supplementary Dataset S4). These results indicate that AIL6 binds almost exclusively to a subset of regions also bound by ANT in stage 3 flowers. This is consistent with AIL6 providing some but not all of the same functions as ANT (Krizek, 2009). A total of 606 unique genes are associated with the 582 overlapping peaks between ANT and AIL6 (Supplementary Dataset S5). Some of the developmental genes bound by both ANT and AIL6 are shown in Table 1.

**Table 1.** Developmental genes associated with both ANT and AIL6 ChIP-Seq peaks

Gene identifier	Gene name	ChIP-Seq binding peak position	Log2 fold change (*ant ail6* or *35S:ANT-GR*)
**Boundary genes**			
AT2G31160	*LSH3/OBO1*	Upstream; downstream	0.90 (*ant ail6*)
AT3G23290	*LSH4*	Upstream	0.85 (*ant ail6*)
AT4G32980	*ATH1*	Overlap start	1.12 (*ant ail6*) –0.238 (4 h *35S:ANT-GR*) –0.258 (4 h *35S:ANT-GR*)
AT3G15170	*CUC1*	Overlap start	–
AT1G76420	*CUC3*	Overlap start	–
AT1G26780	*LOF1*	Upstream	–
**Vascular genes**			
AT2G37590	*DOF2.4/PEAR1*	Upstream; downstream	–
AT5G60200	*DOF5.3/TMO6*	Upstream	0.253 (4 h *35S:ANT-GR*) 0.247 (8 h *35S:ANT-GR*)
AT1G07640	*OBP2*	Downstream	1.15 (*ant ail6*)
AT2G34925	*CLE42*	Upstream	
AT2G27230	*LHW*	Overlap end	
AT1G26600	*CLE9*	Overlap start	
AT5G61480	*PXY*	Upstream	0.67 (*ant ail6*)
AT5G65700	*BAM1*	Downstream	
AT1G19850	*MP*	Upstream	–0.59 (*ant ail6*)
AT5G60690	*REV*	Overlap start	
AT1G19050	*ARR7*	Overlap start	1.25 (*ant ail6*) –0.541 (4 h *35S:ANT-GR*) –0.477 (8 h *35S:ANT-GR*)
AT5G62230	*ERL1*	Upstream; overlap start	–0.76 (*ant ail6*)
**Polarity genes**			
AT5G60690	*REV*	Overlap start	–
AT2G37630	*AS1*	Upstream	–0.235 (4 h *35S:ANT-GR*) –0.175 (8 h *35S:ANT-GR*)
AT3G57130	*BOP1*	Upstream	–0.449 (4 h *35S:ANT-GR*)
AT2G41370	*BOP2*	Upstream	0.65 (*ant ail6*)
AT2G45190	*FIL/YAB1*	Upstream	–0.47 (*ant ail6*)
AT5G16560	*KAN1*	Upstream; inside	–
AT1G32240	*KAN2*	Inside	0.227 (4 h *35S:ANT-GR*) 0.255 (8 h *35S:ANT-GR*)
**Floral organ morphogenesis genes**			
AT3G54340	*AP3*	Overlap start	–2.38 (*ant ail6*)
AT5G20240	*PI*	Overlap start	–2.29 (*ant ail6*)
AT4G18960	*AG*	Inside	–1.26 (*ant ail6*)
AT1G24260	*SEP3*	Inside	0.175 (8 h *35S:ANT-GR*)
AT5G67060	*HEC1*	Upstream	–
AT3G50330	*HEC2*	Upstream	–2.24 (*ant ail6*)
AT2G01940	*SGR5/IDD15*	Downstream	0.320 (4 h *35S:ANT-GR*) 0.497 (8 h *35S:ANT-GR*)
AT1G25250	*IDD16*	Downstream	–

### ANT and AIL6 ChIP-Seq peaks contain DNA-binding motifs for AIL/PLT, BPC, and bHLH transcription factors

**Fig. 4.**
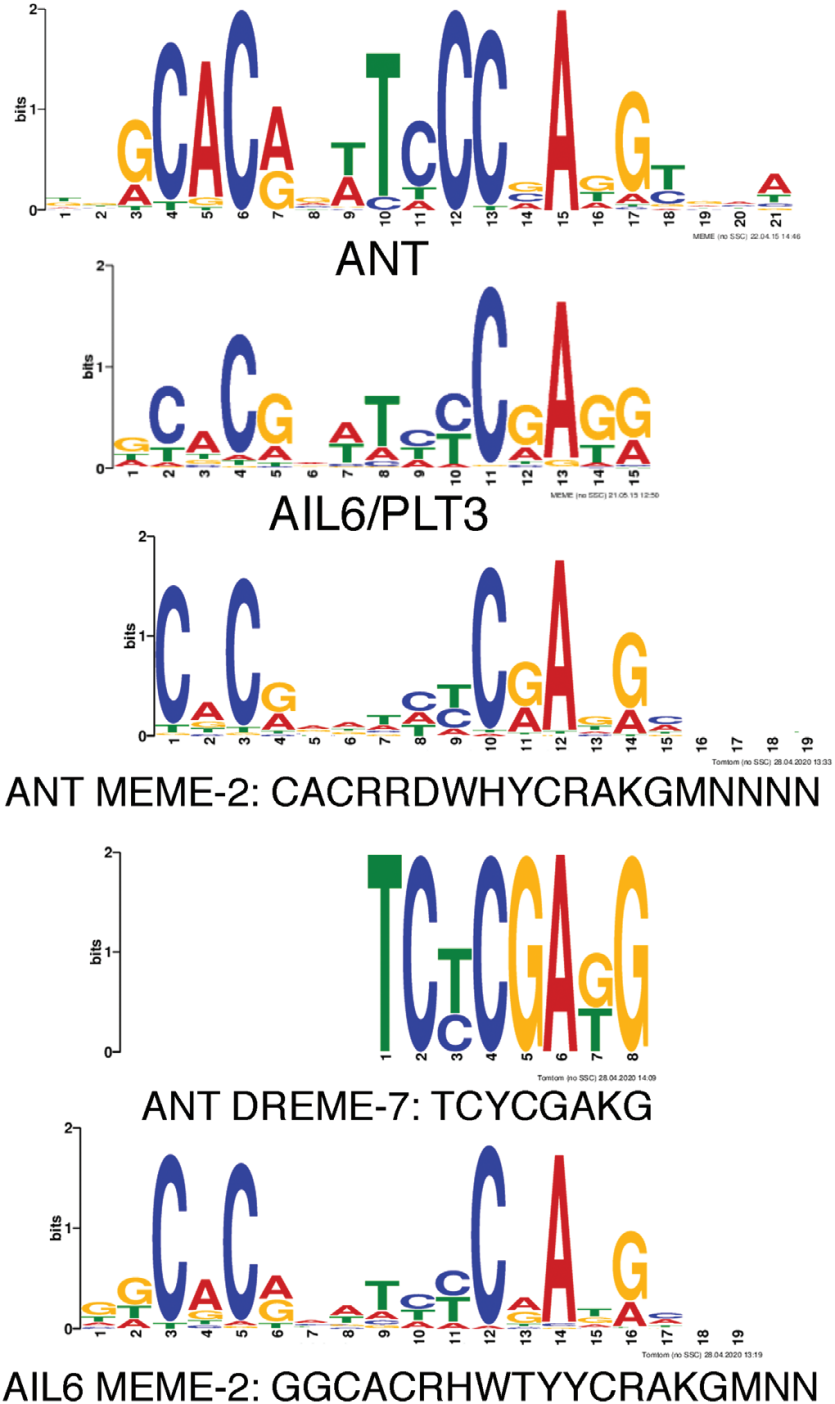
DNA motifs with similarity to ANT- and AIL6-binding motifs are over-represented in ANT and AIL6 ChIP-Seq binding peaks. Top: sequence logos for ANT- and AIL6-binding motifs. Middle: two motifs over-represented in ANT-binding peaks with similarity to the ANT-binding motif are ANT MEME-2 and ANT DREME-7. Bottom: one motif over-represented in AIL6-binding peaks with similarity to the AIL6-binding motif is AIL6 MEME-2.

MEME-ChIP from the MEME Suite was used to perform *de novo* motif discovery on the binding peaks for both ANT and AIL6 (Machanick and Bailey, 2011). This analysis used the DAP-Seq database for motif discovery which contains motifs for several AIL/PLT transcription factors including AIL6 but not ANT (O’Malley *et al.*, 2016). AIL/PLT-binding motifs are fairly long, with a few conserved residues on both ends of the motif and somewhat fewer conserved nucleotides in the center, as shown for AIL6 (Fig. 4). This is less true for the *in vitro* determined ANT-binding motif which consists of more conserved residues throughout the motif (Fig. 4) (Nole-Wilson and Krizek, 2000). For both ANT and AIL6 ChIP-Seq binding peaks, motifs with similarity to those bound by AIL/PLT transcription factors were identified (Table 2). For ANT, these motifs are MEME-2 (CACRRDWHYCRAKGMNNNN) and DREME-7 (TCYCGAKG), and for AIL6 the single motif MEME-2 (GGCACRHWTYYCRAKGMNN) was identified (Table 2; Fig. 4). The ANT MEME-2 and AIL6 MEME-2 motifs are very similar and contain conserved nucleotides toward both ends of the AIL/PLT-like-binding motif, while the DREME-7 motif identified in ANT-binding peaks has similarity to the right half of AIL/PLT-like motifs (Fig. 4).

**Table 2.** MEME-ChIP analysis of ANT and AIL6 ChIP-Seq peaks

ANT motifs	Motif ID	Width	Sites	e-value	Most similar DAP-Seq motif
RRRRARARARARARARARARR	MEME-1	21	689	5.70E-194	BPC5 (BBR/BPC)
CACRRDWHYCRAKGMNNNN	MEME-2	19	355	8.00E-45	AIL7 (AP2/ERF)
AGAGAGM	DREME-1	7	437	1.20E-29	BPC1 (BBR/BPC)
CACRTS	DREME-2	6	686	8.10E-22	AREB3 (bZIP)
CACGTGDCAYDYRCR	MEME-4	15	88	1.50E-11	BIM2 (bHLH)
TCYCGAKG	DREME-7	8	57	1.40E-08	AIL6 (AP2/ERF)
**AIL6 motifs**	**Motif ID**	**Width**	**Sites**	**e-value**	**Most similar DAP-Seq motif**
GGCACRHWTYYCRAKGMNN	MEME-2	19	219	3.20E-186	PLT1 (AP2/ERF)
RRRARARARAGARARAGARG	MEME-1	20	198	1.40E-118	BPC5 (BBR/BPC)
CACGTGDCKTBYKC	MEME-3	14	50	6.00E-13	bHLH74 (bHLH)
AGAGAVA	DREME-1	7	161	3.80E-10	BPC1 (BBR/BPC)
CACGHG	DREME-2	6	103	8.20E-10	bHLH74 (bHLH)

FIMO (Find Individual Motif Occurrences) identified 224 ANT MEME-2 motifs and 298 AIL6 MEME-2 motifs present in ANT/AIL6 overlapping binding peaks (Grant *et al.*, 2011). A total of 185 of the 224 ANT MEME-2 motifs map to the same location as an AIL6 MEME-2 motif with an offset of two nucleotides, because the ANT MEME-2 site lacks the GG nucleotides at positions one and two of the AIL6 MEME-2 motif. This suggests that either ANT or AIL6 can bind to a single DNA sequence that matches both the ANT MEME-2 and AIL6 MEME-2 motifs present within these peaks. The remaining ANT MEME-2 motifs were not separated with any conseved distance from an AIL6 MEME-2 motif.

BARLEY B-RECOMBINANT/BASIC PENTACYSTEINE (BBR/BPC) and basic helix–loop–helix (bHLH) transcription factor-binding motifs were over-represented in both ANT and AIL6 stage 3 binding peaks (Table 2; Supplementary Fig. S1). Previously, we found that binding motifs for these two families of transcription factors were over-represented in ANT-binding peaks identified in stage 6 and 7 flowers (Krizek *et al.*, 2020). BBR/BPC are broadly expressed transcription factors involved in many developmental processes (Monfared *et al.*, 2011). They mediate gene silencing by recruiting Polycomb repressive complexes or other regulatory proteins to GAGA motifs (Simonini *et al.*, 2012; Hecker *et al.*, 2015; Xiao *et al.*, 2017). Binding motifs for basic leucine zipper transcription factors (bZIPs) were also over-represented in ANT-binding peaks (Table 2). These other transcription factors may act in combination with ANT and AIL6 in regulating transcription of target genes.

### Identification of genes bound by ANT and AIL6 and differentially expressed in *ant ail6* or *35S:ANT-GR* inflorescences

Direct targets of a transcription factor are typically defined as genes whose regulatory region is bound by the transcription factor and whose expression is altered in response to changes in the activity of the transcription factor. While ChIP-Seq can identify many hundreds or thousands of transcription factor-binding sites, not all of the genes associated with these sites may be transcriptionally regulated by these binding events. Jointly analyzing ChIP-Seq and RNA-Seq offers the best approach to identify direct target genes. A comparison of the set of genes bound in stage 3 flowers by both ANT and AIL6 (606 genes) with the set of genes that are differentially expressed in *ant ail6* inflorescences (8012 genes) identified an overlap of 175 genes (Fig. 4B) (Krizek *et al.*, 2016).

A second comparison of the genes bound in stage 3 flowers by both ANT and AIL6 (606 genes) with genes that are differentially expressed after induction of ANT activity in *35S:ANT-GR* inflorescences (1195 genes) (Krizek *et al.*, 2020) identified 111 genes that may be direct targets of ANT and AIL6 regulation (Fig. 4C). Within this set of 111 genes, 29 genes are differentially expressed in both the *ant ail6* and *35S:ANT-GR* RNA-Seq datasets (Supplementary Dataset S6). Of these 29 genes, 15 show opposite regulation in *35S:ANT-GR* and *ant ail6* inflorescences (i.e. down-regulated in *35S:ANT-GR* and up-regulated in *ant ail6* or, vice versa, up-regulated in *35S:ANT-GR* and down-regulated in *ant ail6*).

### ANT and AIL6 activate class B and C homeotic genes in stage 3 flowers

**Fig. 5.**
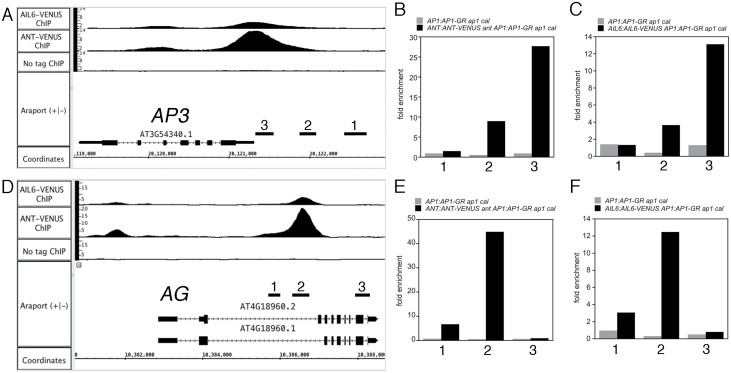
ANT and AIL6 bind directly to regulatory regions associated with the floral organ identity genes *AP3* and *AG*. (A) ChIP-Seq of ANT and AIL6 binding to the *AP3* genomic region. 1, 2, and 3 are the genomic regions tested for binding using ChIP-qPCR in (B) and (C). (B) ChIP-qPCR of ANT binding to *AP3* genomic region 3. (C) ChIP-qPCR of AIL6 binding to *AP3* genomic region 3. (D) ChIP-Seq of ANT and AIL6 binding to the *AG* genomic region. 1, 2, and 3 are the genomic regions tested for binding using ChIP-qPCR in (E) and (F). (E) ChIP-qPCR of ANT binding to *AG* genomic region 2. (F) ChIP-qPCR of AIL6 binding to *AG* genomic region 2.

Included in the set of 175 genes that are both bound by ANT and AIL6 and differentially expressed in *ant ail6* inflorescences are the class B floral homeotic genes *AP3* and *PI*, and the class C floral homeotic gene *AG* (Fig. 5A, D; Table 1; Supplementary Fig. S2A). We confirmed the ChIP-Seq results for *AP3*, *PI*, and *AG* using ChIP-qPCR (Fig. 5B, C, E, F; Supplementary Fig. S2B, C). Expression of these three genes is down-regulated in *ant ail6* inflorescences (Krizek *et al.*, 2016). In addition, *in situ* hybridization had shown previously that *AP3* and *AG* were expressed in fewer cells of stage 3 floral primordia (Krizek, 2009). These results are consistent with the loss of petal, stamen, and carpel identities in *ant ail6* flowers (Krizek, 2009). These data suggest that ANT and AIL6 might directly activate the expression of class B and C floral homeotic genes in young flowers.

**Fig. 6.**
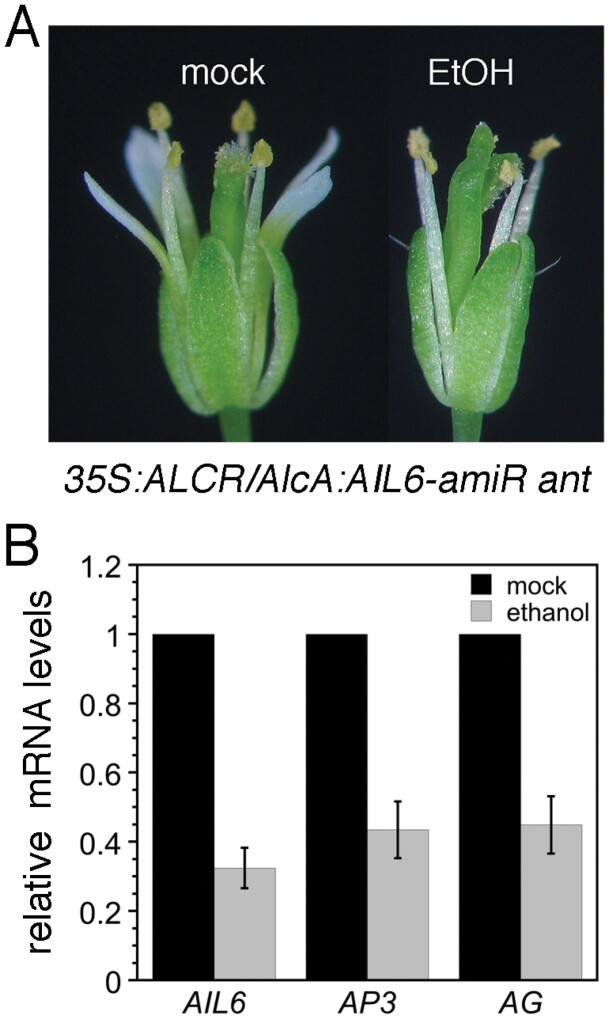
*AP3* and *AG* expression is reduced soon after down-regulation of *AIL6* expression in *35S:ALCR/AlcA:AIL6-amiR ant* inflorescences. (A) Mock- and ethanol (EtOH)-treated *35S:ALCR/AlcA:AIL6-amiR ant* flowers. (B) Expression of *AIL6*, *AP3*, and *AG* is reduced in *35S:ALCR/AlcA:AIL6-amiR ant* inflorescences after an 8 h EtOH treatment. Error bars show the SD.

To investigate whether ANT and AIL6 directly control transcription of *AP3*, *PI*, and *AG*, we examined whether class B and C gene expression responds quickly to changes in ANT and AIL6 activity. We used an ethanol-inducible transgenic line in which *AIL6* expression is down-regulated by an amiRNA in the *ant* mutant background (*35S:ALCR/AlcA:AIL6-amiR ant*; i.e. *AIL6-amiR ant*). After an 8 h ethanol treatment, *AIL6-amiR ant* flowers exhibit a phenotype more severe than *ant*, with loss of petals and partially unfused carpels, suggesting that AIL6 activity is reduced (Fig. 6A). Mock-treated *AIL6-amiR ant* flowers resemble *ant* (Fig. 6A). *AIL6* mRNA levels in the ethanol-treated inflorescences are ~30% of those in mock-treated inflorescences, indicating that the *AIL6-amiR* knocks down *AIL6* expression (Fig. 6B). *AP3* and *AG* mRNA levels but not *PI* mRNA levels are reduced after this 8 h ethanol treatment (Fig. 6B; Supplementary Fig. S2D). These data further support the conclusion that ANT and AIL6 directly activate expression of the class B gene *AP3* and the class C gene *AG* in stage 3 flowers.

No DNA sequences with obvious similarity to ANT- or AIL6-binding motifs are present in *AP3* genomic regions (Nole-Wilson and Krizek, 2000; O’Malley *et al.*, 2016; Krizek *et al.*, 2020). The ANT MEME-2 motif and the AIL6 MEME-2 motif identified here were also not present in these peaks. Thus, is it not clear what DNA sequence is bound by ANT and AIL6 within the *AP3* genomic region. The ANT- and AIL6-binding peaks upstream of *AP3* do overlap the defined PEE core element that is required for early stage 3–5 expression (Lamb *et al.*, 2002). Two partially overlapping sequences with weak similarity to the ANT-binding motif are present near the summit of the ANT and AIL6 ChIP-Seq binding peaks within the *AG* intron (Supplementary Fig. S3A). ANT can bind specifically to this region of the *AG* intron *in vitro* and can activate transcription in yeast through this sequence (Supplementary Fig. S3B, C). For both *AP3* and *AG*, the ANT- and AIL6-binding peaks overlap those of other known regulators of *AP3*, *PI*, and *AG*, including LFY, AP1, AP2, AP3, PI, AG, and SEP3 (Kaufmann *et al.*, 2009, 2010; Yant *et al.*, 2010; Winter *et al.*, 2011; Wuest *et al.*, 2012; Ó’Maoiléidigh *et al*., 2013) (Supplementary Fig. S4A, B).

### Cross-regulation of *AIL* expression may involve direct repression by ANT and AIL6

Previously, we found evidence of cross-regulation among *AIL* gene expression (Krizek *et al.*, 2016). Specifically, we found that *AIL6* mRNA levels are increased in *ant* mutants and that *ANT*, *AIL6*, and *AIL7* mRNA levels are increased in *ant ail6* double mutants (Krizek *et al.*, 2016). Our ChIP-Seq results show that both ANT and AIL6 bind the regulatory regions of *ANT*, *AIL5*, and *AIL6*; ANT also binds to the regulatory region of *AIL7* (Supplementary Fig. S5). This suggests that the observed repression of *AIL* expression by ANT and AIL6 may be mediated by direct binding of ANT and AIL6 to *AIL* regulatory regions.

As seen with the floral homeotic genes, ANT- and AIL6-binding peaks in these regulatory regions overlap those of other floral regulators, including LFY, AP1, AP3, PI, and AG (Kaufmann *et al.*, 2010; Winter *et al.*, 2011; Wuest *et al.*, 2012; Ó’Maoiléidigh *et al*., 2013) (Supplementary Fig. S5). *ANT* and *AIL6* expression in floral anlagen occurs with similar timing to *LFY* expression, and LFY was previously shown to bind to *ANT* and *AIL6* genomic regions (Winter *et al.*, 2011). Thus, LFY may contribute to *ANT* and *AIL6* expression in floral primordia. In contrast, *AP1*, *AP3*, *PI*, and *AG* are expressed later in flower development than *ANT* and *AIL6* and thus would only contribute to maintenance or refinement of *ANT* and *AIL6* expression at later stages of development, if they play a role at all.

### ANT and AIL6 directly regulate organ growth genes

*ANT* and *AIL6* both positively contribute to floral organ growth, although *AIL6* cannot completely substitute for *ANT* in this role as *ant* single mutants produce smaller floral organs (Elliott *et al.*, 1996; Klucher *et al.*, 1996). Several growth-regulating genes are bound by both ANT and AIL6 in our ChIP-Seq experiments and exhibit differential expression in either *ant ail6* double mutants or dex-treated *35S:ANT-GR* inflorescences. These include the growth repressor *BIG BROTHER* (*BB*), and the growth-promoting genes *ROTUNDIFOLIA3* (*ROT3*), *ANGUSTIFOLIA3/GRF-INTERACTING FACTOR 1* (*AN3/GIF1*), and *XYLOGLUCAN ENDOTRANSGLUCOSYLASE/HYDROLASE9* (*XTH9*) (Supplementary Fig. S6). *BB* expression was elevated in *ant ail6* double mutants, *ROT3* expression was reduced in *ant ail6* double mutants, and *AN3/GIF1* and *XTH9* expression was up-regulated by induction of ANT-GR activity (Krizek *et al.*, 2016, 2020). *AN3/GIF1* and *XTH9* were previously identified as likely direct targets of ANT regulation as their regulatory regions are bound by ANT in stage 6/7 flowers (Krizek *et al.*, 2020).

**Fig. 7.**
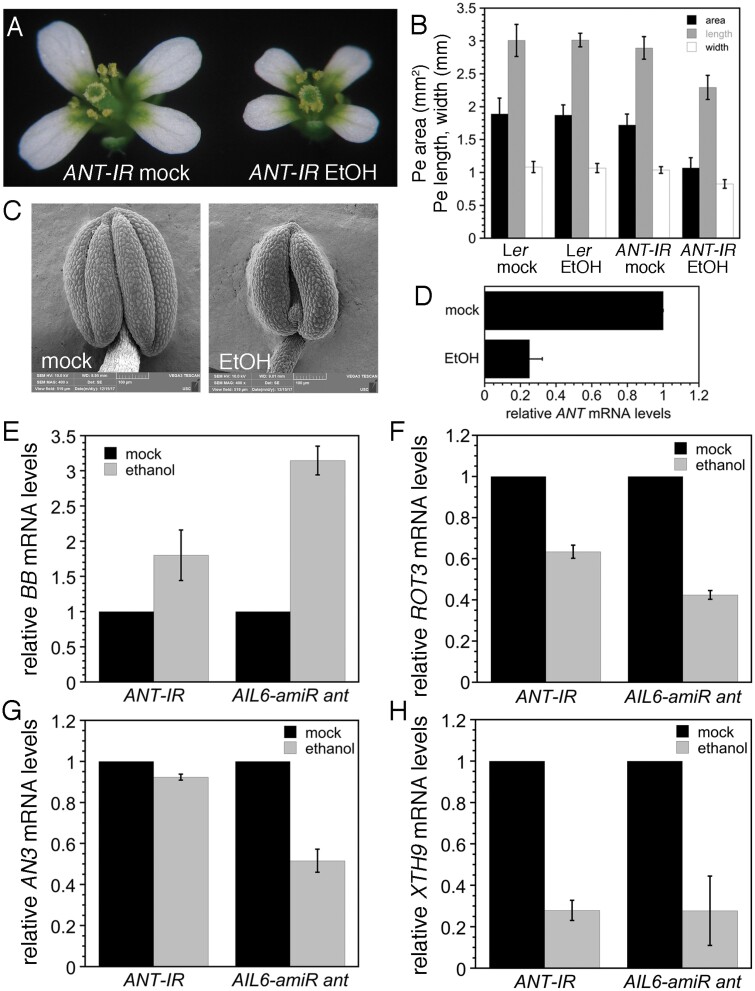
Expression of growth-regulatory genes is altered after down-regulation of *ANT* expression in *35S:ALCR/AlcA:ANT-IR* inflorescences and after down-regulation of *AIL6* expression in *35S:ALCR/AlcA:AIL6-amiR ant* inflorescences. (A) Mock- and ethanol (EtOH)-treated *35S:ALCR/AlcA:ANT-IR* flowers. (B) Petal area, length, and width measurements for mock- and EtOH-treated L*er* and *35S:ALCR/AlcA:ANT-IR* flowers. Between 10 and 20 petals from different flowers were measured for each genotype and treatment. (C) Stamen anther of a mock-treated (left) and ethanol-treated (right) *35S:ALCR/AlcA:ANT-IR* flower. (D) Expression of *ANT* is reduced in *35S:ALCR/AlcA:ANT-IR* inflorescences after a 24 h EtOH treatment. (E) *BB* expression is up-regulated in *35S:ALCR/AlcA:ANT-IR* and *35S:ALCR/AlcA:AIL6-amiR ant* inflorescences after ethanol treatment. (F) *ROT3* expression is down-regulated in *35S:ALCR/AlcA:ANT-IR* and *35S:ALCR/AlcA:AIL6-amiR ant* inflorescences after ethanol treatment. (G) *AN3* expression is slightly down-regulated in *35S:ALCR/AlcA:ANT-IR* inflorescences and more severely down-regulated in *35S:ALCR/AlcA:AIL6-amiR ant* inflorescences. (H) *XTH9* expression is down-regulated in *35S:ALCR/AlcA:ANT-IR* and *35S:ALCR/AlcA:AIL6-amiR ant* inflorescences after ethanol treatment. Error bars in all panels show the SD.

We further investigated whether these genes are likely to be direct targets of ANT and AIL6 regulation by examining their expression in response to down-regulation of *AIL6* in the *ant* mutant background and down-regulation of *ANT* alone. To down-regulate *ANT* expression, we generated an RNAi line that utilizes an ethanol-inducible inverted repeat transgene (*35S:ALCR/AlcA:ANT-IR*; i.e. *ANT-IR*). After ethanol treatment, *ANT-IR* flowers produce smaller petals and anthers with two locules similar to *ant* mutants (Fig. 7A–C). After a 24 h ethanol treatment, *ANT* mRNA levels are reduced to ~25% of that of the mock-treated plants (Fig. 7D). *BB* expression was increased 1.8-fold in ethanol-treated *ANT-IR* and 3.1-fold in ethanol-treated *AIL6-amiR ant* inflorescences, suggesting that both ANT and AIL6 contribute to repression of *BB* expression (Fig. 7E). After ethanol induction, *ROT3* expression levels were 63% of the levels seen in mock-treated *ANT-IR* and 42% of the levels seen in mock-treated *AIL6-amiR* inflorescences (Fig. 7F). *AN3/GIF1* expression was slightly reduced in *ANT-IR* inflorescences and reduced ~2-fold in *AIL6-amiR* inflorescences (Fig. 7G). This suggests that for *AN3/GIF1* regulation, AIL6 can largely compensate for loss of ANT. After ethanol induction, *XTH9* expression was reduced ~3-fold in both *ANT-IR* and *AIL6-amiRNA ant* inflorescences (Fig. 7H).

### ANT and AIL6 directly regulate genes with roles in vascular development

**Fig. 8.**
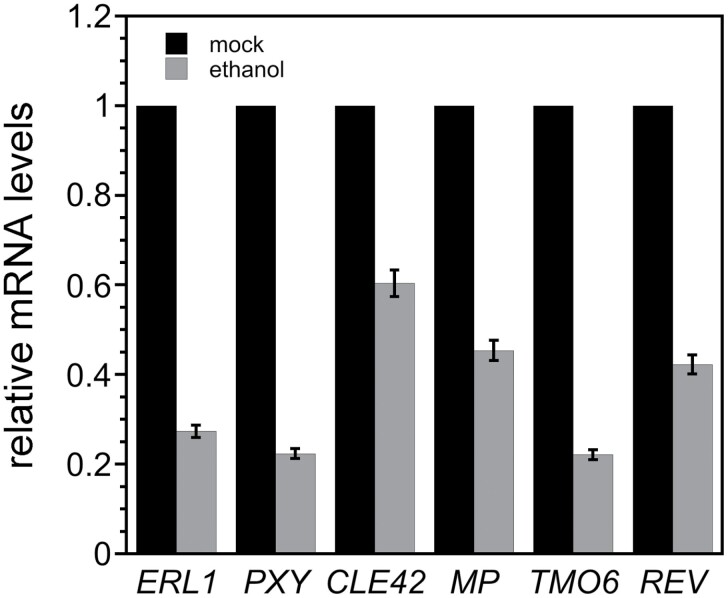
Vascular development genes are expressed at reduced levels after down-regulation of *AIL6* expression in *35S:ALCR/AlcA:AIL6-amiR ant* inflorescences. *ERL1*, *PXY*, *CLE42*, *MP*, *TMO6*, and *REV* mRNA levels are reduced after a 8 h ethanol treatment of *35S:ALCR/AlcA:AIL6-amiR* inflorescences.

A number of genes that function in vascular development were present among the radial patterning genes bound by both ANT and AIL6 (Table 1; Supplementary Fig. S7). Because *ANT* and *AIL6* are expressed in the procambium of young inflorescence tissue and flowers, it is possible that some of these genes are direct targets of ANT and AIL6 regulation (Elliott *et al.*, 1996; Krizek, 2015). *ant* mutants in both Arabidopsis and maize show defects in leaf vascular pattern (Kang *et al.*, 2007; Liu *et al.*, 2020). In addition, *ant ail6* double mutants show more severe reductions in leaf vein density and complexity than *ant* single mutants (Krizek, 2009). *ERECTA-LIKE1* (*ERL1*), *PHLOEM INTERCALATED WITH XYLEM/TDIF RECEPTOR* (*PXY/TDR*), and *CLAVATA3/ESR-RELATED 42* (*CLE42*) mRNA levels were reduced to 27, 22, and 60% of the mock-treated levels (Fig. 8). *MP*, *TARGET OF MONOPTEROS* (*TMO6*), and *REVOLUTA (REV)* mRNA levels were reduced to 45, 27, and 42% of the mock-treated levels (Fig. 8).

## Discussion

Our data indicate that the partially overlapping roles of ANT and AIL6 in flower development are a consequence of these transcription factors regulating many of the same target genes. The more important role of ANT in floral organogenesis as compared with AIL6 appears to result from ANT regulating additional genes that are not targets of AIL6 regulation. In addition, at most of these genomic sites, ANT exhibits higher occupancy than AIL6, which may lead to a more significant effect on transcriptional regulation at these sites. Higher occupancy by ANT may be a consequence of greater amounts of ANT protein in these stage 3 flowers as compared with AIL6 rather than intrinsic differences in the DNA binding affinities of ANT and AIL6. *ANT* mRNA levels in wild-type inflorescences are ~8-fold higher than those of *AIL6* (Han and Krizek, 2016). In addition, endogenous AIL6 protein may have competed with AIL6–VENUS for binding to genomic sites in the ChIP-Seq experiment. Previous work has shown that AIL6 can compensate for loss of ANT function when AIL6 is expressed under the control of the *ANT* promoter at levels similar to *ANT* mRNA levels (Han and Krizek, 2016).

GO analyses on genes associated with ANT and AIL6 ChIP-Seq peaks suggest that these transcription factors regulate a number of different processes during early stages of flower development. In particular, terms associated with floral meristem development (meristem initiation, maintenance of meristem identity, and floral meristem determinacy) and floral organ development (polarity specification, formation of plant organ boundary, radial pattern formation, and floral organ morphogenesis) were identified (Fig. 2). Many of the GO terms identified here match those identified in our earlier ANT ChIP-Seq experiment using stage 6/7 flowers (Krizek *et al.*, 2020). However, several more terms associated with the initiation and patterning of floral organ primordia were identified here, including floral organ formation, formation of plant organ boundary, radial pattern formation, and floral organ morphogenesis (Fig. 2). This suggests additional roles for ANT and AIL6 in boundary specification and radial pattern formation during floral organogenesis.

We show that ANT and AIL6 directly activate the class B gene *AP3* and the class C gene *AG* in stage 3 flowers. ANT and AIL6 bind to *AP3* and *AG* regulatory regions (Fig. 5), expression of *AP3* and *AG* is reduced soon after down-regulation of AIL6 activity in the *ant* mutant background (Fig. 6), and *AP3* and *AG* are expressed in fewer cells in stage 3 flowers (Krizek, 2009). The regulation of *AG* expression by ANT appears to be complex as *ANT* acts with *APETALA2* (*AP2*) to repress *AG* expression in second whorl cells, although it is not known if this regulation is direct (Krizek *et al.*, 2000). Thus, ANT and AIL6 may directly activate *AG* expression in third and fourth whorl cells while repressing (directly or indirectly) *AG* expression in second whorl cells. How broadly expressed ANT and AIL6 activate *AP3* specifically in second and third whorl cells and *AG* specifically in third and fourth whorl cells is not clear. It is possible that ANT and AIL6 act with region-specific cofactors as has been shown previously for LFY (Lee *et al.*, 1997; Busch *et al.*, 1999; Lenhard *et al.*, 2001; Lohmann *et al.*, 2001; Lamb *et al.*, 2002; Liu *et al.*, 2009). Another question still to be answered is what is the DNA sequence bound by ANT and AIL6 within the *AP3* promoter. No obvious AIL/PLT-like-binding motif was identified within the ChIP-Seq binding peaks. Two overlapping sequences with weak similarity to the ANT-binding motif are present within the *AG* intron and bound by ANT *in vitro* (Supplementary Fig. S3). This weak similarity suggests that there may be flexibility in the DNA sequences recognized by ANT. These sequences are located near the LFY- and WUSCHEL (WUS)-binding sites within the *AG* intron (Busch *et al.*, 1999; Lohmann *et al.*, 2001). The ANT and AIL6 ChIP-Seq binding peaks within both *AP3* and *AG* overlap with those of other floral regulatory genes (Supplementary Fig. S4). Thus, these genomic regions appear to contain binding sites for multiple transcription factors that may act cooperatively to regulate transcription. In particular, ANT and AIL6 may act in combination with other transcriptional regulators such as LFY, SEP3, AP1, and AP2 to control the spatial and temporal expression of *AP3* and *AG*.

ANT and AIL6 contribute to other aspects of floral organogenesis including growth; this appears to involve the regulation of both growth-promoting and growth-repressing genes. ANT and AIL6 directly bind to a region near the TSS of the growth repressor *BB* (Supplementary Fig. S6A), and *BB* expression is increased in *ant ail6* double mutant inflorescences (Fig. 7E) (Krizek *et al.*, 2016). *bb* mutants produce larger flowers than the wild type, while overexpression of *BB* results in smaller flowers (Disch *et al.*, 2006). *BB* is expressed throughout young stage 1–4 flowers, similar to *ANT* expression in young flowers and overlapping with *AIL6* (Elliott *et al.*, 1996; Nole-Wilson *et al.*, 2005; Disch *et al.*, 2006). Previous work has shown that transgenic *bb* plants containing a genomic copy of *BB* with a deletion in an upstream region corresponding to the location of the ANT and AIL6 ChIP-Seq binding peaks produced smaller flowers than the wild type (Breuninger and Lenhard, 2012). The reduced size of these flowers suggests that these transgenic plants have higher levels of *BB* expression than the wild type and that this region contains a negative *cis*-regulatory element bound by ANT and AIL6 that represses transcription of *BB*. BB is an E3 ubiquitin ligase that ubiquitinates and activates the protease DA1 and is subsequently cleaved by DA1 (Disch *et al.*, 2006; Dong *et al.*, 2017). DA1 limits the duration of the cell proliferation phase of organ growth by cleaving the deubiquitylase UBP15 and two TEOSINTE BRANCHED 1/CYCLOIDEA/PCF transcription factors, TCP15 and TCP22 (Dong *et al.*, 2017). While BB protein levels are regulated by DA1, ANT and AIL6 are the first identified potential regulators of *BB* transcription.

ANT and AIL6 also bind to regions associated with three growth-promoting genes, *ROT3*, *AN3*, and *XTH9* (Supplementary Fig. S6B–D). Previously we showed that ANT binds to the regulatory regions of *AN3* and *XTH9* in stage 6/7 flowers and that both of these genes are activated upon dex induction of ANT-GR activity (Krizek *et al.*, 2020). Here we show that ANT and AIL6 bind to these genomic regions in stage 3 flowers. Furthermore, reduced expression of *ROT3*, *AN3*, and *XTH9* upon down-regulation of *AIL6* in the *ant* mutant background provides additional evidence that ANT and AIL6 directly regulate the expression of these genes (Fig. 7F–H). This suggests that ANT and AIL6 regulate a variety of growth-regulating pathways as ROT3 is a cytochrome P450 acting in brassinosteroid biosynthesis, AN3 is a transcriptional co-activator that works with GROWTH REGULATING FACTOR (GRF) transcription factors, and XTH enzymes modify xyloglucan in the cell wall (Kim *et al.*, 1998; Rose *et al.*, 2002; Kim and Kende, 2004).

Several of the radial patterning genes bound by ANT and AIL6 are associated with vascular development (Supplementary Fig. S7). ANT and AIL6 are expressed in procambial cells of the young inflorescence stem and developing flowers, although their function in these cells is not known (Elliott *et al.*, 1996; Krizek, 2015). Transcriptional profiling of inflorescence stems detected the highest expression of *AIL6* and *ANT* in the cambium, although there is expression of each gene in other vascular cell types (Shi, 2020). To further investigate the potential role of ANT and AIL6 in development of the procambium in young floral buds, we measured the expression of six genes with known roles in regulating cell division of procambial cells. *ERL1*, *PXY*, *CLE42*, *MP*, *TMO6*, and *REV* RNA levels were reduced in ethanol-treated *35S:ALCR/AlcA:AIL6-amiR ant* inflorescences, suggesting that these genes might be directly regulated by ANT and AIL6 (Fig. 8). ERL1 and PXY are leucine rich-repeat receptor-like kinases (LRR-RLKs) that act in parallel signaling pathways to maintain procambial cell identity (Fisher and Turner, 2007; Uchida and Tasaka, 2013; Wang *et al.*, 2019). EPIDERMAL PATTERNING FACTOR LIKE (EPFL) peptides perceived by ERL1 in phloem cells promote cell proliferation and/or inhibit differentiation of the adjacent cambial cells (Uchida and Tasaka, 2013). TRACHEARY ELEMENT DIFFERENTIATION INHIBITORY FACTOR (TDIF) peptides, including CLE42, bound by PXY receptors in the procambium promote division of the procambium, inhibit xylem differentiation, and organize vascular patterning (reviewed in Etchells *et al.*, 2016)] In addition, ANT and AIL6 may contribute to the regulation of procambium proliferation through MP. One pathway mediated by MP is through the PEAR transcription factor TMO6 (Schlereth *et al.*, 2010; Miyashima, 2019). TMO6 produced in phloem precursor cells moves into adjacent procambial cells to promote their division (Miyashima, 2019). PEAR activity is restricted to cambial cells on the phloem side by HD-ZIP transcription factors including REV (Miyashima, 2019). Thus, our data suggest that ANT and AIL6 regulate procambium identity and proliferation through multiple pathways.

In summary, our work provides new insights into the roles of ANT and AIL6 in the initiation and development of floral organs from the floral meristem. We have identified direct targets of ANT and AIL6 regulation that mediate their roles in the establishment of floral organ identity, promotion of floral organ growth, and development of the procambium. Additional work is needed to further elaborate the roles of ANT and AIL6 in these processes and to determine whether other genes identified here are direct targets of ANT and AIL6 that may mediate their roles in additional aspects of floral organogenesis.

## Supplementary data

The following supplementary data are available at *JXB* online.

Table S1. Primers used in this study.

Fig. S1. DNA motifs with similarity to BBR/BPC- and bHLH-binding motifs are over-represented in ANT and AIL6 ChIP-Seq binding peaks.

Fig. S2. ANT and AIL6 bind directly to regulatory regions associated with the floral organ identity gene *PI*, but *PI* expression is not altered after down-regulation of *AIL6* expression in *35S:ALCR/AlcA:AIL6-amiR ant* inflorescences.

Fig. S3. ANT binds to the *AG* intron *in vitro* and can activate transcription through this binding site in yeast.

Fig. S4. ANT- and AIL6-binding peaks within *AP3* and *AG* regulatory regions overlap those of other floral regulators.

Fig. S5. ANT- and AIL6-binding peaks within *ANT*, *AIL6*, and *AIL7* regulatory regions overlap those of other floral regulators.

Fig. S6. ANT and AIL6 ChIP-Seq binding peaks in *BB*, *ROT3*, *AN3*, and *XTH9* genomic regions.

Fig. S7. ANT and AIL6 ChIP-Seq binding peaks in *ERL1*, *PXY*, *CLE42*, *MP*, *TMO6*, and *REV* genomic regions.

Dataset S1. Genes associated with ANT or AIL6 stage 3 ChIP-Seq binding peaks

Dataset S2. GO terms over-represented in genes associated with ANT stage 3 ChIP-Seq binding peaks

Dataset S3. GO terms over-represented in genes associated with AIL6 stage 3 ChIP-Seq binding peaks

Dataset S4. AIL6 peaks that lack an overlapping ANT peak

Dataset S5. Genes associated with overlapping ANT and AIL6 stage 3 ChIP-Seq binding peaks

Dataset S6. Genes associated with overlapping ANT and AIL6 stage 3 ChIP-Seq binding peaks and differentially expressed in *ant ail6* and *35S:ANT-GR* inflorescences (2, 4, and 8 h)

erab223_suppl_Supplementary_File001

erab223_suppl_Supplementary_File002

erab223_suppl_Supplementary_File003

erab223_suppl_Supplementary_File004

erab223_suppl_Supplementary_File005

erab223_suppl_Supplementary_File006

erab223_suppl_Supplementary_File007

## Data Availability

ChIP-Seq sequences are available from the Sequence Read Archive (https://www.ncbi.nlm.nih.gov/sra) accession number PRJNA685489. Version-controlled source codes used to process and analyze data are available from https://bitbucket.org/krizeklab. Sequence alignments and coverage graphs are available for interactive visualization within the Integrated Genome Browser (Nicol *et al.*, 2009). To view the data in IGB, readers may download and install the software from https://bioviz.org. Once installed, datasets from the study can be opened within IGB by selecting the latest *Arabidopsis thaliana* genome and then choosing the ChIP-Seq folder within the Available Data Sets section of the Data Access Panel.
