# Supplementary material for: AINTEGUMENTA and AINTEGUMENTA-LIKE6 directly regulate floral homeotic, growth, and vascular development genes in young Arabidopsis flowers
Source: J Exp Bot. 2021 May 20;72(15):5478–93. doi: 10.1093/jxb/erab223 (PMC8318262; doi:10.1093/jxb/erab223)
Supplement: erab223_suppl_Supplementary_File001 [file erab223_suppl_supplementary_file001.pdf]

Supplementary Figures and Tables

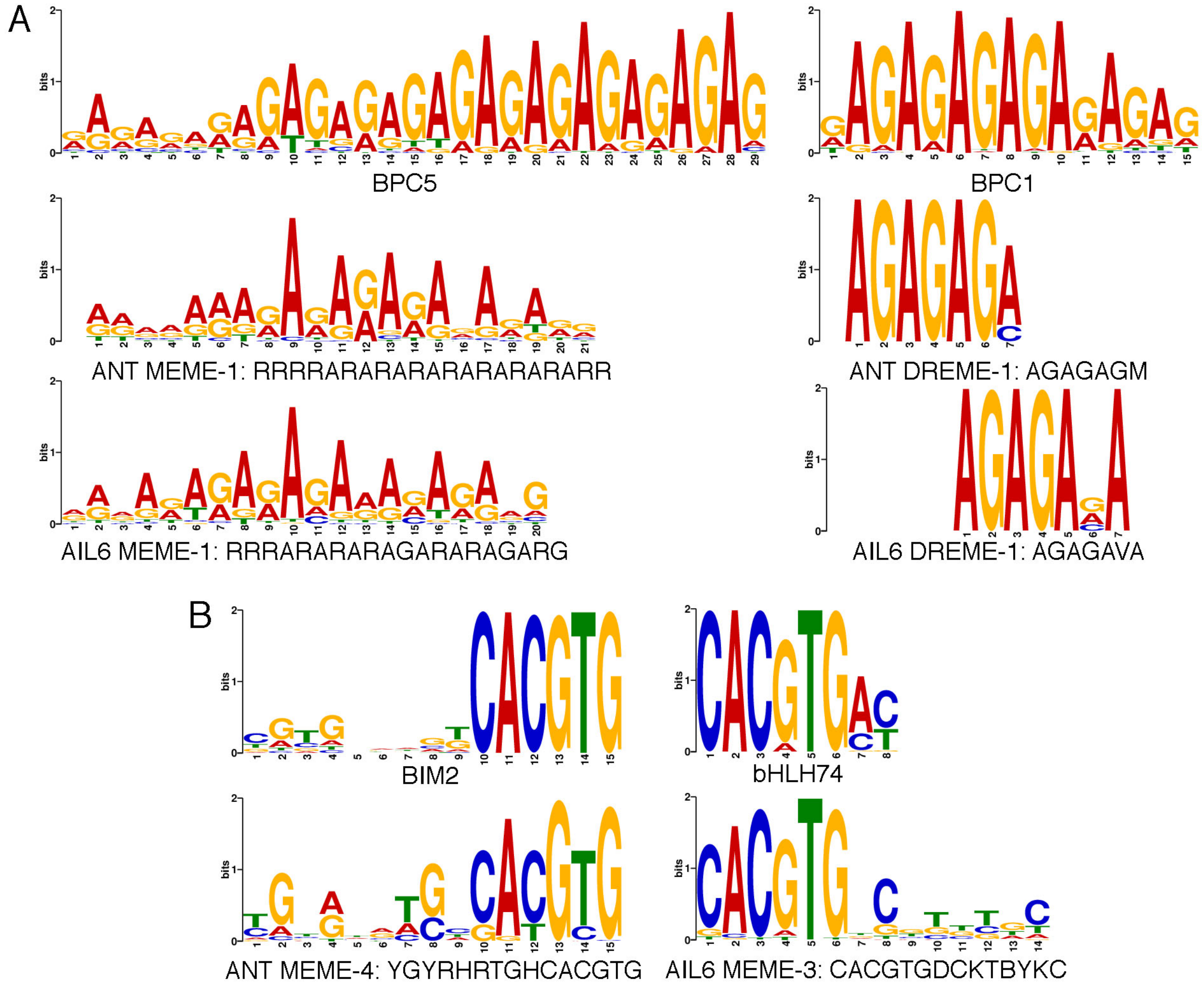


**Supplementary Figure S1. DNA motifs with similarity to BBR/BPC and bHLH binding motifs are over-represented in ANT and AIL6 ChIP-Seq binding peaks.** A. Sequence logos for the BPC5 and BPC1 binding motifs and motifs overrepresented in ANT binding peaks (ANT MEME-1 and ANT DREME-1) and AIL6 binding peaks (AIL6 MEME-1 and AIL6 DREME-1) that have similarity to the BPC motif. B. Sequence logos for the BIM2 and bHLH74 binding motifs and motifs overrepresented in ANT binding peaks (ANT MEME-4) and AIL6 binding peaks (AIL6 MEME-3) that have similarity to the bHLH motifs.

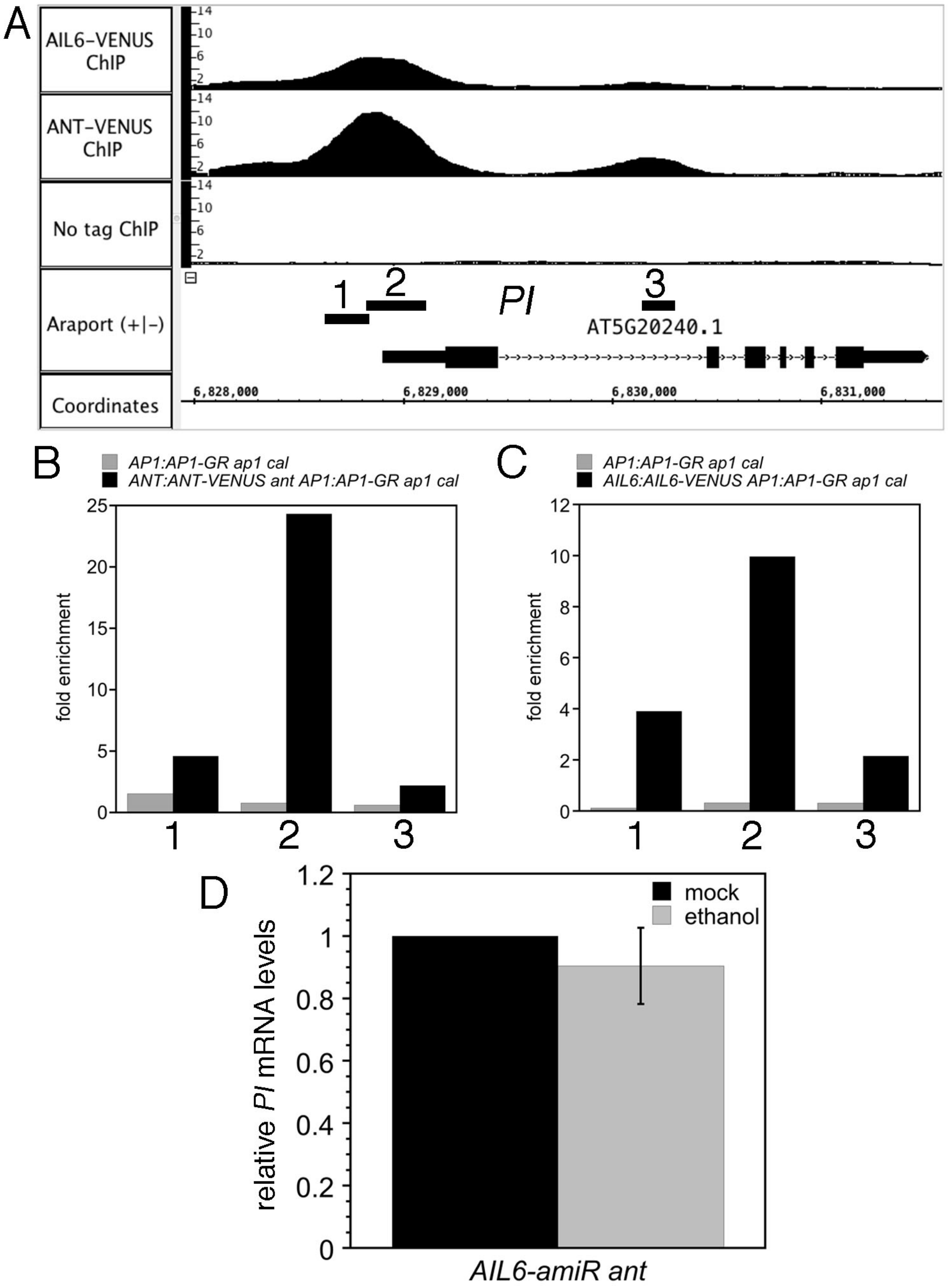


**Supplementary Figure S2. ANT and AIL6 bind directly to regulatory regions associated with the floral organ identity gene *PI,* but *PI* expression is not altered after downregulation of *AIL6* expression in *35S:ALCR/AlcA:AIL6-amiR ant* inflorescences.** A. ChIP-Seq of ANT and AIL6 binding to *PI* genomic region. 1, 2, and 3 are genomic regions tested for binding using ChIP-qPCR in B and C. B. ChIP-qPCR of ANT binding to *PI* genomic region 2. C. ChIP-qPCR of AIL6 binding to *PI* genomic region 2. D. Expression of *PI* is not reduced in *35S:ALCR/AlcA:AIL6-amiR ant* inflorescences after an eight-hour ethanol treatment.

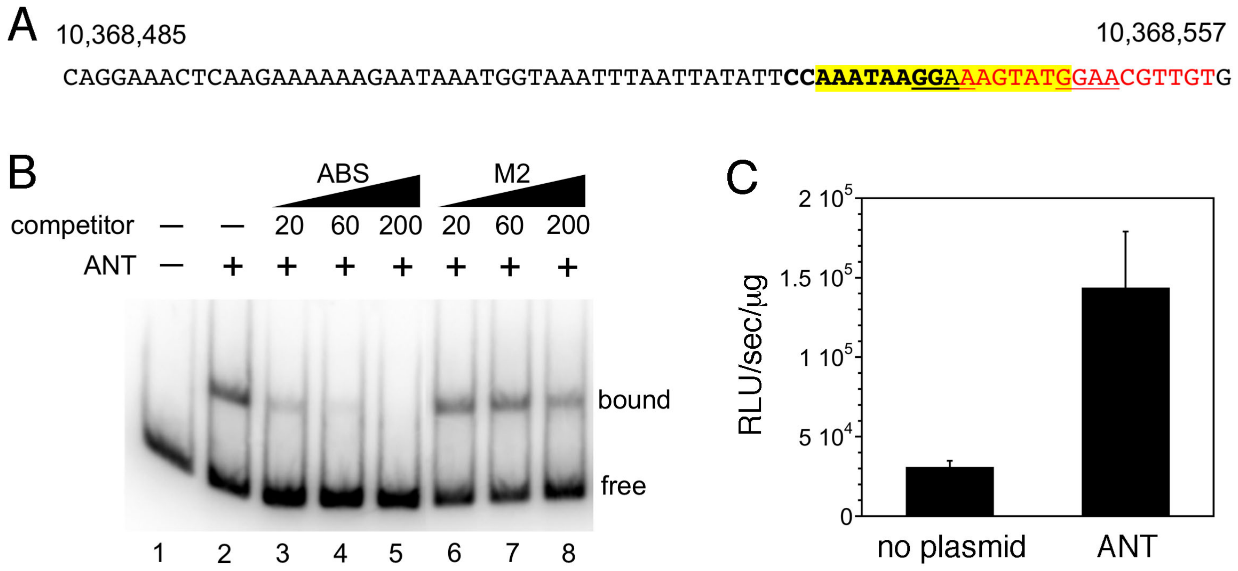


**Supplementary Figure S3. ANT binds to the *AG* intron *in vitro* and can activate transcription through this binding site in yeast.** A. Two overlapping sequence within the *AG* intron (genomic coordinates 10,368,485-10,368,557) with weak similarity to the ANT binding motif. One motif is shown in red and the other motif is highlighted in yellow. A central GGAA (reverse complement TTCC) in these two motifs is underlined. An overlapping CArG box is shown in bold type. LFY binding sites are located at 10,368,608-10,368,614 and 10,368,655-10,368,661. WUS binding sites are located at 10,368,603-10,368,609 and 10,368,663-10,368,669. B. Gel mobility shift of ANT binding to a probe corresponding to the *AG* intron sequence shown in A. The binding is competed away by addition of the consensus ANT binding site (ABS) but not by addition of a mutated version of this site (M2) (Nole-Wilson and Krizek, 2000). C. ANT can activate transcription in a yeast strain containing the *lacZ* reporter gene under the control of this *AG* intron sequence and the TATA portion of the *CYC1* gene.

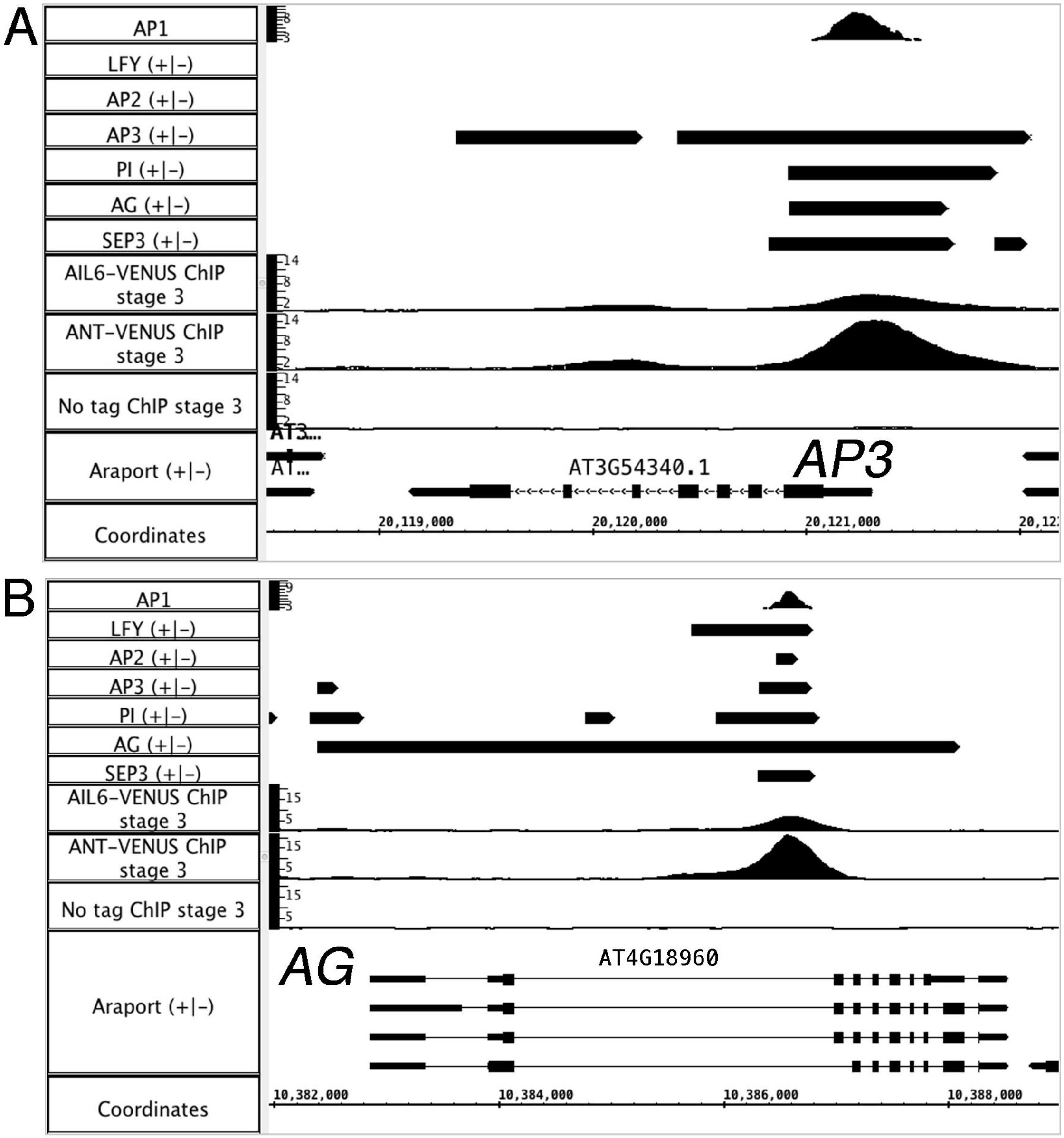


**Supplementary Figure S4. ANT and AIL6 binding peaks within *AP3* and *AG* regulatory regions overlap those of other floral regulators.** A. ANT and AIL6 binding peaks overlap those of AP1, AP3, PI, AG and SEP3 in the *AP3* promoter. B. ANT and AIL6 binding peaks overlap those of LFY, AP1, AP2, AP3, PI, AG and SEP3 in the *AG* intron.

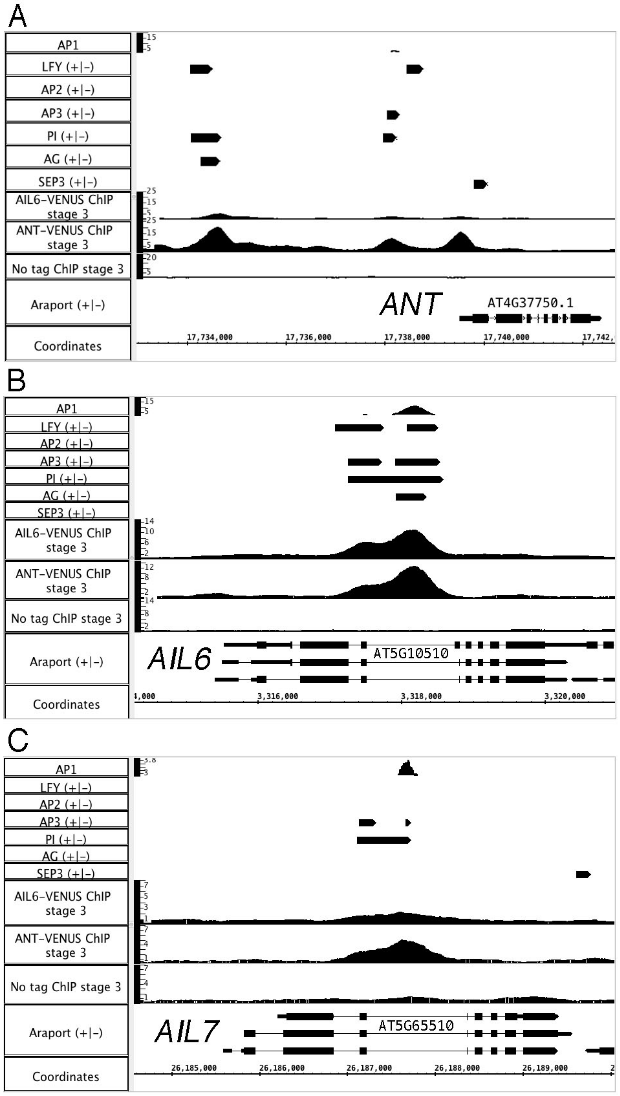


**Supplementary Figure S5. ANT and AIL6 binding peaks within *ANT, AIL6* and *AIL7* regulatory regions overlap those of other floral regulators.** A. ANT and AIL6 binding peaks overlap those of LFY, AP1, AP3, PI, and AG in the *ANT* promoter. B. ANT and AIL6 binding peaks overlap those of LFY, AP1, AP3, PI, and AG in the *AIL6* intron. C. ANT and AIL6 binding peaks overlap those of AP1, AP3, and PI in the *AIL7* intron.

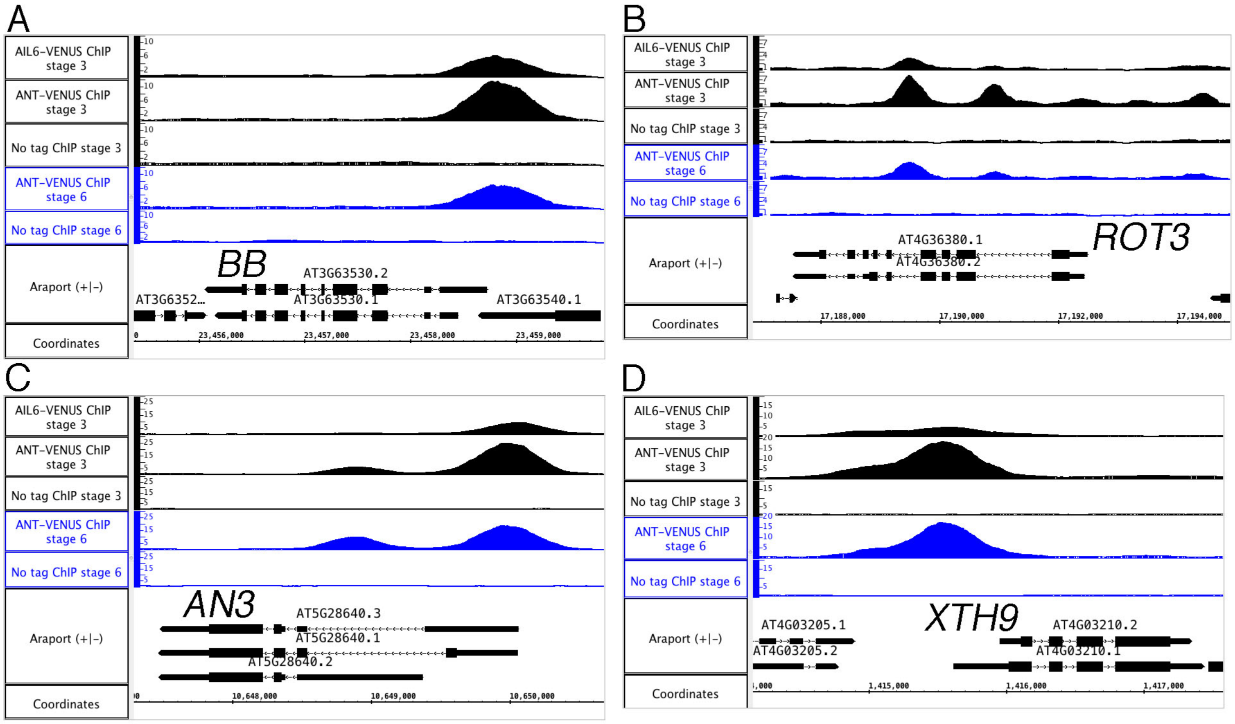


**Supplementary Figure S6. ANT and AIL6 ChIP-Seq binding peaks in *BB*, *ROT3*, *AN3* and *XTH9* genomic regions.** A. Binding of ANT and AIL6 in stage 3 flowers and ANT in stage 6/7 flowers to a region near the TSS of *BB*. B. Binding of ANT and AIL6 in stage 3 flowers and ANT in stage 6/7 flowers to regions inside *ROT3*. C. Binding of ANT and AIL6 in stage 3 flowers and ANT in stage 6/7 flowers to a region overlapping the TSS of *AN3*. D. Binding of ANT and AIL6 in stage 3 flowers and ANT in stage 6/7 flowers to a region near the TSS of *XTH9*.

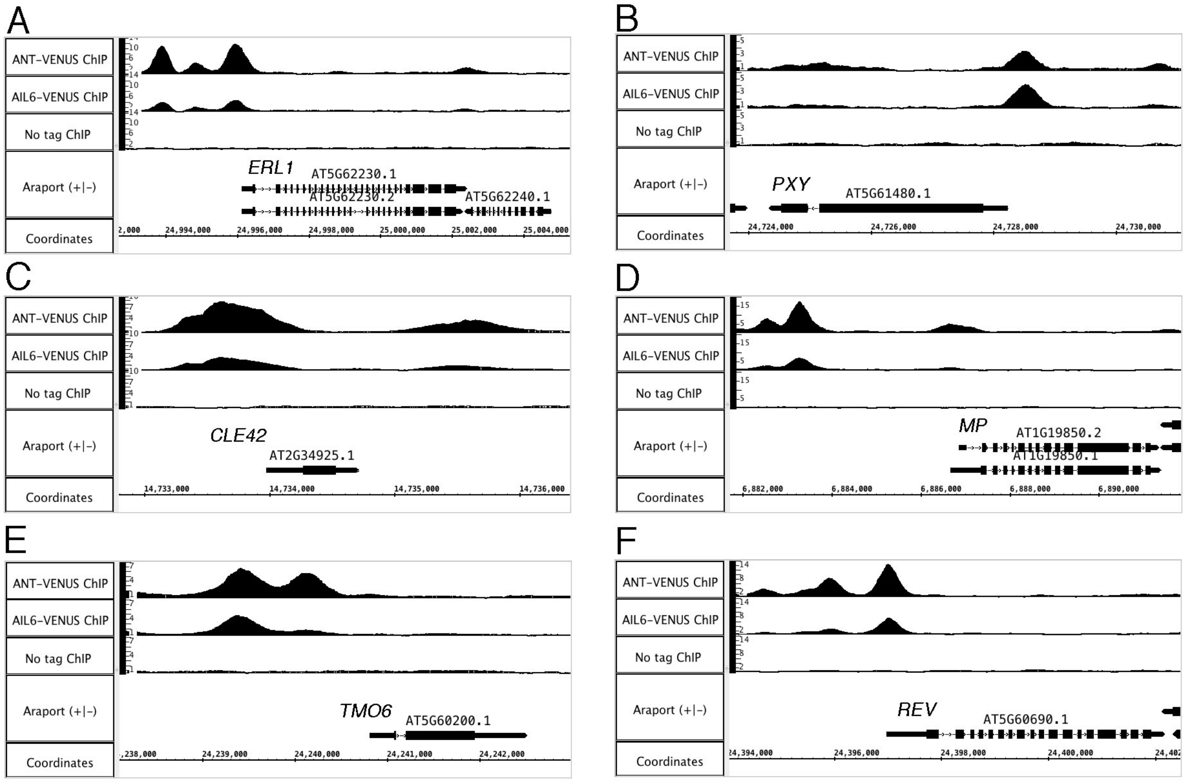


**Supplementary Figure S7. ANT and AIL6 ChIP-Seq binding peaks in *ERL1*, *PXY, CLE42*, *MP, TMO6,* and *REV* genomic regions.** A. Binding of ANT and AIL6 in stage 3 flowers to regions upstream and near the TSS of *ERL1*. B. Binding of ANT and AIL6 in stage 3 flowers to region upstream of *PXY*. C. Binding of ANT and AIL6 in stage 3 flowers to a region upstream of *CLE42*. D. Binding of ANT and AIL6 in stage 3 flowers to a region upstream of *MP*. D. Binding of ANT and AIL6 in stage 3 flowers to a region upstream of *MP*. E. Binding of ANT and AIL6 in stage 3 flowers to a region upstream of *TMO6.* F. Binding of ANT and AIL6 in stage 3 flowers to a region near the TSS of *REV*.

**Supplementary Table S1. Primers used in this study**

| **ChIP-qPCR primers** | **Primer sequence (5' to 3')** |
|---|---|
| *AP3* | |
| fragment 1 | AP3 Pro7: CGATCATACGGCTGGGTGAT |
| | AP3 Pro8: AAGGCATTCCCCGTATCTGC |
| fragment 2 | AP3 Pro3: TGATTTGATGGACTGTTTGGAG |
| | AP3 Pro4: TTTGGATTAATCGTCACTTCCA |
| fragment 3 | AP3 Pro5: CATCGATGTCCGTTGATTTA |
| | AP3 Pro6: TTTGGTGGAGAGGACAAGAGA |
| *AG* | |
| fragment 1 | AG INT-1: AGAGAGTCCCACGTGATTACTT |
| | AG INT-2: AATCTTGCGCTCAATTCCAACC |
| fragment 2 | AG INT-5: TGGGTACTGAGAGGAAAGTGAG |
| | AG INT-6: TGGTCTGAACATGTCTAGGGTT |
| fragment 3 | AG A3F: AGACCAAACCGCTCTCCAGT |
| | AG A3R: TTGCTTGCTCAACCCAATTC |
| *PI* | |
| fragment 1 | PI Pro1: GCTAAATAAAAGTTTGACCACATTTC |
| | PI Pro2: CACTCTTTGCATGTGATTGATG |
| fragment 2 | PI Pro3: TGCAAAGAGTGTTCATTAAGCAA |
| | PI Pro4: GAACAAGAAGAGGAGCATTGGT |
| fragment 3 | P1 I-1: AGGGTAATAAGCATACAGAGGC |
| | PI I-2: GCATATAACACACACACACACACTG |
| **RT-qPCR primers** | |
| *AIL6* | RTAIL6-4: CGAGTTGCTGGGAACAAAG |
| | RTAIL6-5: TCATACGCTTCAGCTGCTTC |
| *AP3* | AP3-F: CGAATGCAAGAAACCAAGAGG |
| | AP3-R: GAATGTCAAGCTCGTCCAAC |
| *AG* | AG-F: GTTCTTTGTGATGCTGAAGTCG |
| | AG-R: TGTACCTCTCAATAGTCCCTTTTAC |
| *PI* | PI-F: AATGAGAAGATGATGGCGGAG |
| | PI-R: CATTCCTCTTGCGTTGCTTG |
| *ANT* | RTANT-7: AAGGAAGAGCAGTGGTTTCTC |
| | RTANT-8: ACTCTACCAATCCGTGCTTG |
| *BB* | BB-F: AAATCGGAGACACAGATGAGC |
| | BB-R: GATCAATGTCATCTTGCCAGG |
| *AN3* | AN3-RT1: CCAAGTGTGCATAGCCAGTA |
| | AN3-RT2: GTTGCTGTTGAGTCGCTTG |
| *XTH9* | XTH9-F: TGGGCTATGGATCATTGTGTC |
| | XTH9-R: TGATTCAAACCCAGCTCCAG |
| *ERL1* | ERL1-F: GTTCATGTGCAGCCTTGAATC |
| | ERL1-R: CAGATAAGTCAAGCTCCCGAG |
| *PXY* | PXY-F: AAGCCAAAGAGGAAGACAGTAG |
| | PXY-R: ACCAACATCAACACTACAAACATC |
| *CLE42* | CLE42-F: GAGAGGTTTAGAGTTCGGCG |

| | |
|---|---|
| | CLE42-R: TGGGACTCCATGTTCATTGG |
| *TMO6* | TMO6-F: AAGCCAAAGAGGAAGACAGTAG |
| | TMO6-R: ACCAACATCAACACTACAAACATC |
| *REV* | REV-F: GCTTAGAGATGCGAATAGTCCTG |
| | REV-R: CAGTTCCTGTAGCCTTGGATAG |
| *MP* | MP-F: CTTCTCACAAGGTCATAGCGAG |
| | MP-R: AACTGAGATGGAAGGTTCGG |
| *AT5G15710* | RTFbox-1: TTTCGGCTGAGAGGTTCGAGT |
| | RTFbox-2: GATTCCAAGACGTAAAGCAGATCAA |
| **Cloning primers** | |
| *ANT-IR* | ANTIR-5: CTCGAGGGATCCATGAAGTCTTTTTGTGATAATG |
| | ANTIR-6: GGTACCATCGATTCGTCTCAAACCCAACGCTTGT |
